# Computational Tools for Hydrogen–Deuterium Exchange Mass Spectrometry Data Analysis

**DOI:** 10.1021/acs.chemrev.4c00438

**Published:** 2024-10-31

**Authors:** Michele Stofella, Antonio Grimaldi, Jochem H. Smit, Jürgen Claesen, Emanuele Paci, Frank Sobott

**Affiliations:** † School of Molecular and Cellular Biology, Faculty of Biological Sciences, University of Leeds, LS2 9JT Leeds, United Kingdom; ‡ Astbury Centre for Structural Molecular Biology, University of Leeds, LS2 9JT Leeds, United Kingdom; § Dipartimento di Fisica e Astronomia, Universita’ di Bologna, 40127 Bologna, Italy; ∥ Department of Microbiology and Immunology, Rega Institute for Medical Research, Laboratory of Molecular Bacteriology, KU Leuven, 3000 Leuven, Belgium; ⊥ Epidemiology and Data Science, Vrije Universiteit Amsterdam, 1081 HV Amsterdam, The Netherlands

## Abstract

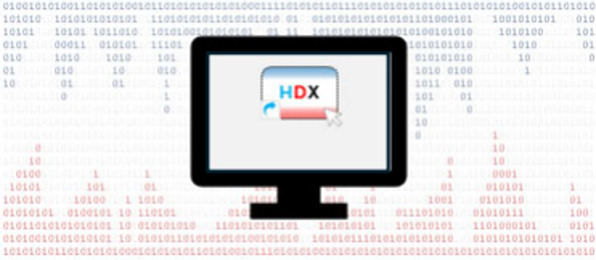

Hydrogen–deuterium exchange (HDX) has become a pivotal method for investigating the structural and dynamic properties of proteins. The versatility and sensitivity of mass spectrometry (MS) made the technique the ideal companion for HDX, and today HDX-MS is addressing a growing number of applications in both academic research and industrial settings. The prolific generation of experimental data has spurred the concurrent development of numerous computational tools, designed to automate parts of the workflow while employing different strategies to achieve common objectives. Various computational methods are available to perform automated peptide searches and identification; different statistical tests have been implemented to quantify differences in the exchange pattern between two or more experimental conditions; alternative strategies have been developed to deconvolve and analyze peptides showing multimodal behavior; and different algorithms have been proposed to computationally increase the resolution of HDX-MS data, with the ultimate aim to provide information at the level of the single residue. This review delves into a comprehensive examination of the merits and drawbacks associated with the diverse strategies implemented by software tools for the analysis of HDX-MS data.

## 1 Introduction

Proteins are the most important gears in the engine of life. Since the seminal work by Anfinsen in 1960, scientists have wondered how their linear sequence of amino acids folds into a defined three-dimensional structure, how these structures change upon binding, and how they maintain health and cause disease. High-resolution snapshots of protein structures can be captured by X-ray crystallography, NMR spectroscopy, or electron microscopy (EM), while their dynamic behavior in solution is harder to probe. Hydrogen bonding is one of the defining aspects of a protein’s structure (or lack thereof), but equally important for how it interacts with the surrounding solvent. One unique feature of proteins is the exchange of their amide hydrogens with hydrogens in solution.^1^ “Proteins continuously emit signals in the language of hydrogen exchange”,^2^ and understanding how to detect and interpret these signals is a unique opportunity to harness protein design.

When diluted into a deuterated buffer, the amide hydrogens of the protein spontaneously exchange with deuterium in solution.^1^ The phenomenon is referred to as hydrogen–deuterium exchange (HDX). In the case of fully unstructured proteins, the rate of exchange depends on chemical properties of the buffer (pH, temperature, ionic strength) on one side, and on the amino acid’s effective p*K*_a_ (determined by its side chain and its direct neighbors). When a protein acquires its native structure, hydrogen bonding and solvent accessibility lower the rate of exchange by means of “protection”, and HDX measures this perturbed rate of exchange, thereby informing on the protein’s structural and dynamic properties.^2^ Measuring the isotopic exchange in proteins posed a technical challenge. In its early years, HDX was measured using an ultracentrifugation procedure;^3^ later, by infrared^4^ or UV^5^ spectroscopy. These techniques have low “spatial resolution”: they cannot monitor the exchange at a residue-level, but only the global exchange of the protein (i.e., the summed exchange of labile sites); yet they cannot determine the overall extent of deuterium incorporation very accurately either. The popularity of HDX increased with the advent of two-dimensional NMR.^6^ Hydrogen and deuterium have different spins (hydrogen has spin 1/2, while deuterium has spin 1); leveraging the decrease of ^1^H NMR signal upon deuteration in an HSQC spectrum,^7^ HDX-NMR can monitor the exchange of individual labeled residues (high spatial resolution) but is limited to the study of small proteins (<40–50 kDa)^8^ and requires larger amounts of sample as well as ^15^N labeling. In the last 30 years, HDX coupled with mass spectrometry (MS) has been established as a viable alternative.^9^ The versatility of the technique^10^ and recent technological advancements^11^ led to the generation of large amounts of data, and today the technique needs computational tools for an automated analysis and for retrieving more detailed and statistically accurate information from the raw data.^12^

**Figure 1:**
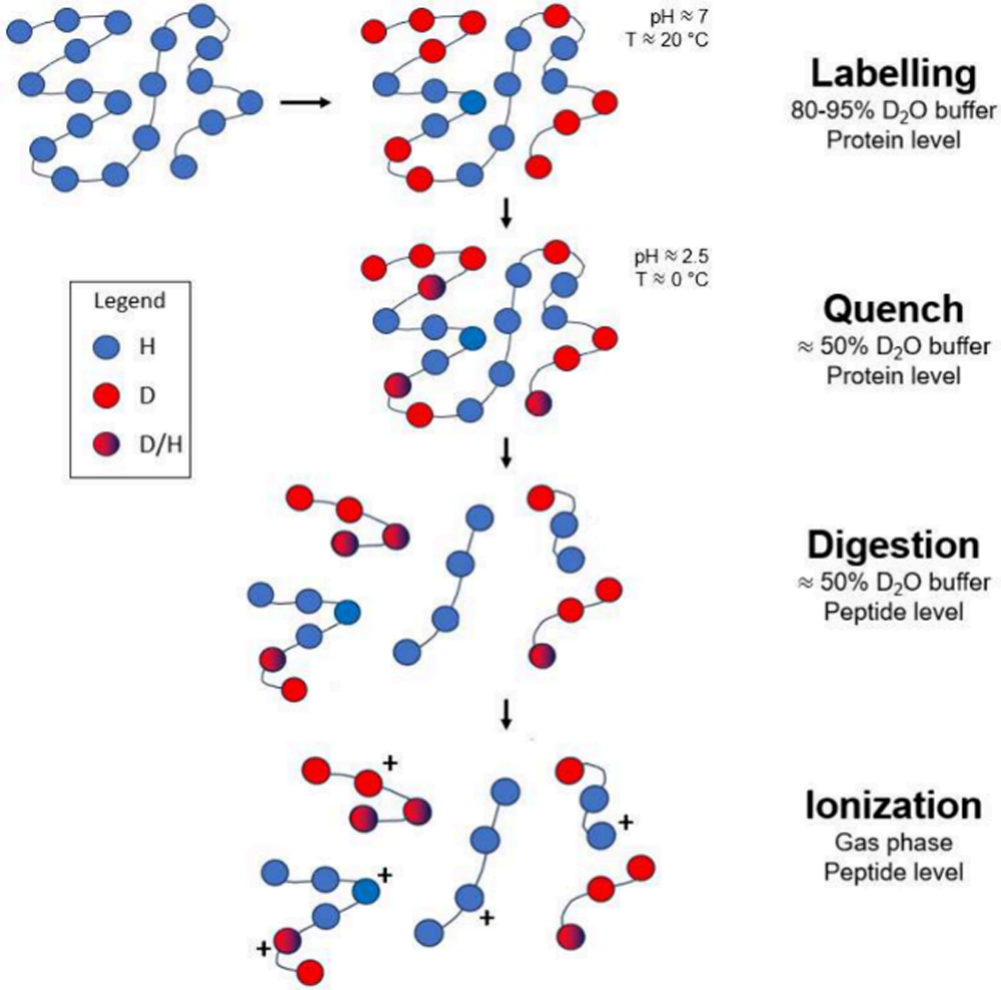
Typical experimental workflow of an HDX-MS experiment: (1) labeling: the undeuterated protein (blue) is diluted in a 80–95% deuterated buffer where the HDX occurs at the protein level for times ranging from milliseconds to hours; deuterated residues are shown in red; (2) quench: the exchange, still occurring at the protein level, is minimized by lowering the temperature (to ∼0 °C) and the pH (to ∼2.5), back-exchange can occur at the protein level (blue/red beads); (3) digestion: the protein is digested, from this point forward-exchange and back-exchange (red-blue circles) occur at the peptide level; and (4) ionization: the proteolytic peptides are ionized and eluted in the mass spectrometer.

HDX-MS measures the mass increase of a protein caused by deuteration (Figure 1).^9,13^ The protein (or complex) is first equilibrated in a suitable biochemical buffer at desired pH, ionic strength and temperature. Continuous H/D exchange starts with dilution into deuterated buffer at a typical ratio of between 1:5 to 1:20 (buffers are generally 80–95% deuterated) and labeling occurs for a variable amount of time. Labeling times generally range from 10s of seconds to hours, but recent technological developments gave access to the millisecond scale,^14−16^ which is crucial to probe the fast exchange of highly dynamic regions and intrinsically disordered proteins,^16^ as well as unstructured peptides^17^ (these are highly valuable for fundamental studies, e.g., to study how H/D exchange is dependent on the buffer conditions). HDX can be monitored at the level of the intact protein (*global* HDX); it is worth noting here that global HDX-MS has been applied to study structured oligonucleotides^18^ and a software, OligoR (not reviewed here), has been developed to analyze these data.^19^ In order to obtain higher spatial resolution (*local* HDX), a “bottom-up” approach is generally implemented: the protein is digested, and the mass spectra of the proteolytic peptides are acquired. While measuring the mass shifts of the intact peptides yields data on the incorporation of deuterium per peptide, MS/MS fragmentation using collision-induced dissociation (CID) is used to confirm the sequence of the peptide without deuteration, but it scrambles hydrogen and deuterium within peptides and is therefore not useful for determining of exchange sites at the single amino acid (residue) level. Before digestion, the exchange must be quenched at low pH (∼2.5) and temperature (∼0 °C) to minimize *back-exchange*, which corresponds to a partial loss of deuterium label. Pepsin is the most used enzyme for protein digestion in HDX-MS experiments because it is active at acidic pH, although other enzymes have been used (such as the fungal proteases XIII and XVIII,^20^ nepenthesin,^21^*Aspergillus niger* prolyl endoprotease,^22^ rice field eel pepsin and aspergillopepsin^23^). These enzymes cleave the protein into peptides, producing nonpredictable yet reproducible patterns of overlapping peptides; the use of multiple enzymes can increase the spatial resolution, which is determined by the digestion pattern. The proteolytic peptides are separated by rapid reversed-phase liquid chromatography (LC) with a gradient time of ∼10 min (possibly holding the column close to 0 °C), ionized with electrospray ionization (ESI), and eluted into the mass spectrometer. Alternative experimental setups for local HDX-MS, not covered in this review, include the fragmentation of the intact labeled protein (“top-down”) or of the proteolytic peptides (“middle-down”) using electron capture dissociation (ECD), electron transfer dissociation (ETD),^24^ or ultraviolet photodissociation (UVPD).^25^

The raw data of the peptide-level experiment comprises the time evolution of the mass spectra of proteolytic peptides of the protein, i.e. their mass shifts. A comprehensive tool for HDX-MS data analysis would (1) identify a list of proteolytic peptides assigned to mass spectra in the raw control (undeuterated) data, (2) assign peaks in the labeled (deuterated) raw spectra of the identified peptides, (3) identify peptides showing a bimodal spectrum (see section 4.3), (4) calculate the mass increase of each peptide, (5) correct for back-exchange, (6) increase the spatial resolution, ideally to residue level (protection factor analysis), and (7) localize and quantify statistically significant differences in the uptake pattern of two (or more) experimental conditions (differential analysis). Steps 1, 2, and 4 are generally conducted using vendor-specific software tools (namely PLGS and DynamX for Waters instruments, BioPharma Finder and HDExaminer for Thermo Fisher Scientific instruments), and the results are then exported to perform further analysis. Back in 2006, HX-Express^26^ was one of the first software tools for HDX-MS data analysis. Since then, several platforms have been developed, such as HDX workbench,^27^ Hydra,^28^ Hexicon,^29^ and ExMS^30^ that have been previously reviewed.^12^ In response to the recommendations for performing, interpreting and reporting HDX-MS experiments published by the international community in 2019,^9^ several methods have been implemented with the goal of providing a standard and comprehensive framework for data visualization and differential analysis. Moreover, stand-alone computational methods have been developed to tackle the most common challenges provided by HDX-MS data, such as corrections for back-exchange, deconvolution of EX1/EX2 kinetics, and protection factor analysis.

The purpose of this paper is to review the recent tools (both commercial and open-source) available for the analysis of continuous labeling, *local* HDX-MS data. First, we evaluate the capability of *comprehensive* software (by comprehensive, we mean a tool ideally able to cover all 7 points mentioned above) of providing a standardized framework for qualitative data visualization and quantitative data analysis for differential experiments (when two or more experimental conditions are compared). Most biochemical experiments have this differential nature, as they compare two or more states of a protein (e.g., mutation, ligand binding, or free against complex). In this common scenario, the data analysis workflow is divided into two parts: a commercial instrument-dependent software is first used to preprocess the experimental data, then a third-party open-source software is used for statistical data analysis. In most scenarios, this analysis is sufficient to answer the research question. Here, we particularly focus on more advanced tools where much more information contained in the data can be extracted. We review and discuss stand-alone programs implementing unique features for “nonstandard” analysis, such as automated peptide search (section 4.1), multimodal analysis (section 4.2), and protection factor analysis (section 4.4). The software and methods reviewed in this paper are listed in Table 1. Note that the figures in this paper have not been created by one of the reviewed methods but by our own Python scripts.

**Table 1:** List of Software Tools and Methods Reviewed in This Paper^a^

Software	Access to Raw Data			
	Automated Peptide Search (section 4.1)	Multimodal Analysis (section 4.2)	Differential Analysis (section 4.3)	High Resolution HDX-MS (section 4.4)
Claesen et al.^31^			+	
DECA^32^			+	+
deMix^33^	+	+		
Deuteros 2.0^34^			+	
ExMS2^35,36^	+	+		+
ExPfact^37,38^				+
HaDeX^39^			+	
HD-eXplosion^40^			+	
HX-Express^41^	+	+		
Hdflex^42^			+	+
HDXAnalyzer^43^			+	
HDXModeller^44^				+
Hdxstats^45^			+	+
*HDX-Workbench*(27)	+	+	+	+
HR-HDXMS^46^				+
Mass Spec Studio^47−50^	+	+	+	
MEMHDX^51^			+	
*Protein Metric (Dotmatics)*	+	+	+	+
PyHDX^52^				+
ReX^53^			+	+
Saltzberg et al.^54^				+

a An updated list of software, publications and other resources for HDX-MS data analysis is available at the following link: https://github.com/hadexversum/HDX-MS-resources. Commercial software are shown in italic.

## 2 Theoretical Background

When a protein is diluted in a solution containing deuterium oxide (D_2_O), its amide hydrogens spontaneously exchange with deuterium (D). It is fair to say that all the hydrogens (H) of the protein are exchanging. However, the labeling time scales that can be probed with an HDX-MS experiment range from milliseconds to hours. In the light of this, carbon-bound aliphatic and aromatic hydrogens exchange far too slowly to be detected, while side chain acidic and basic hydrogens and polar −OH, −SH, and −NH_2_ groups exchange too fast, and therefore they rapidly back-exchange into hydrogen during the LC-MS analysis and are lost before detection.^55,56^

Amide hydrogens are fully “exchange competent” (“open” state NH_op_) when they are surface exposed and not engaged in secondary structure (i.e., they do not form hydrogen bonds other than with water). Some residues are structurally protected against exchange (“closed” state NH_cl_), but local fluctuations (defined by the opening and closing rates *k*_op_ and *k*_cl_) can expose them to solvent-enabled deuteration and subsequently undergo exchange to form the deuterated state (ND).^2^ As a consequence, HDX of a single amide hydrogen can be modeled as a two-step process (Linderstrøm-Lang model):^1^
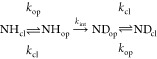
1

The intrinsic exchange rate *k*_int_ corresponds to the exchange rate of the residue in a completely unfolded structure. It depends on chemical properties of the buffer (pH, temperature and ionic strength) as well as the amino acid itself and the neighboring residues.^17,57−59^ HDX-MS is a kinetic experiment, with the ultimate goal of determining the rates of exchange defined in eq 1.

The exact analytical solution for the model in eq 1 is a double exponential.^60^ Under the so-called native approximation for a mostly folded peptide backbone (*k*_op_ ≪ *k*_cl_, i.e. the amide residue is mostly in the closed, protected state) and the EX2 regime (*k*_int_ ≪*k*_cl_, i.e. the exchange is slow compared to the local structural dynamics), the deuteration of a single residue (d)—considering the deuterated residue either in the NH_op_ or ND_cl_ state—can be approximated as a single exponential:
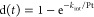
2

The pseudo (pre)equilibrium constant *P* ≡ *k*_cl_/*k*_op_ is known as protection factor and encodes dynamic properties of the protein:^61^ several microscopic models have been developed aiming to connect the structure of a protein to its protection factors; the most known model, often addressed as “phenomenological model”, describes the protection factor of a residue as the linear combination of heavy contacts (i.e., the number of atoms in the proximity of the amide not belonging to neighboring residues in the primary sequence) and hydrogen bonds.^62,63^ These models have already been reviewed by Devaurs et al.^64^ and will not be discussed here.

Under denaturing conditions and for intrinsically disordered proteins, the amide backbone is largely exposed and the exchange kinetics may follow the so-called EX1 regime (occurring when *k*_cl_ ≪ *k*_int_).^65^ The deuterium uptake of a single residue can be approximated to occur in a single step with a rate *k*_op_:
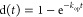
3

The presence of EX1 or EX2 kinetics (or their coexistence, known as EXX kinetics) can be discriminated in the raw HDX-MS data by the emergence of a bimodal pattern of the isotopic distribution in the mass spectrum of the peptide (see section 4.3).^66^ However, this bimodal pattern is not guaranteed to occur in EX1 conditions. Indeed, when EX1 conditions are met, the exact analytical solution of the Linderstrøm-Lang model (eq 1) provides fast exchange kinetics per residue but no explanation for the bimodal pattern for the peptide. The explanation of the bimodal pattern stands in the cooperativity between residues, which is exclusive to peptide-level HDX-MS data and cannot be monitored by NMR experiments: under EX1 conditions, the probabilities of closing (*k*_cl_) and exchanging (*k*_int_) are such that, if subsets of residues open cooperatively, it is likely that most of (or all) the residues exchange, forming the second, fully exchanged population of the distribution.^67^ Other factors, discussed in section 4.2, may also lead to a bimodal pattern.

## 3 Connecting Theory and HDX-MS Data

HDX-MS experiments usually detect the deuterium uptake of a protein through its proteolytic peptides. Before performing any kind of analysis, preprocessing of the raw mass spectra is required to identify these peptides from the LC-MS/MS runs. This peptide search is performed on a digested control sample (without deuterium labeling). Identification of proteolytic peptides follows similar procedures as bottom-up proteomics, albeit for nontryptic peptides in the case of HDX, and is generally performed using commercial software included with the instrument: PLGS and DynamX for Waters instruments, BioPharma Finder and HDExaminer for Thermo Fisher Scientific/Trajan (section 3.1). Next, the quality of the isotopic envelopes of each peptide (and charge state) is checked manually to verify assignments and eliminate false identifications. The major drawbacks of the semiautomated peptide search provided by these software and alternative strategies are discussed in section 4.1.

**Figure 2:**
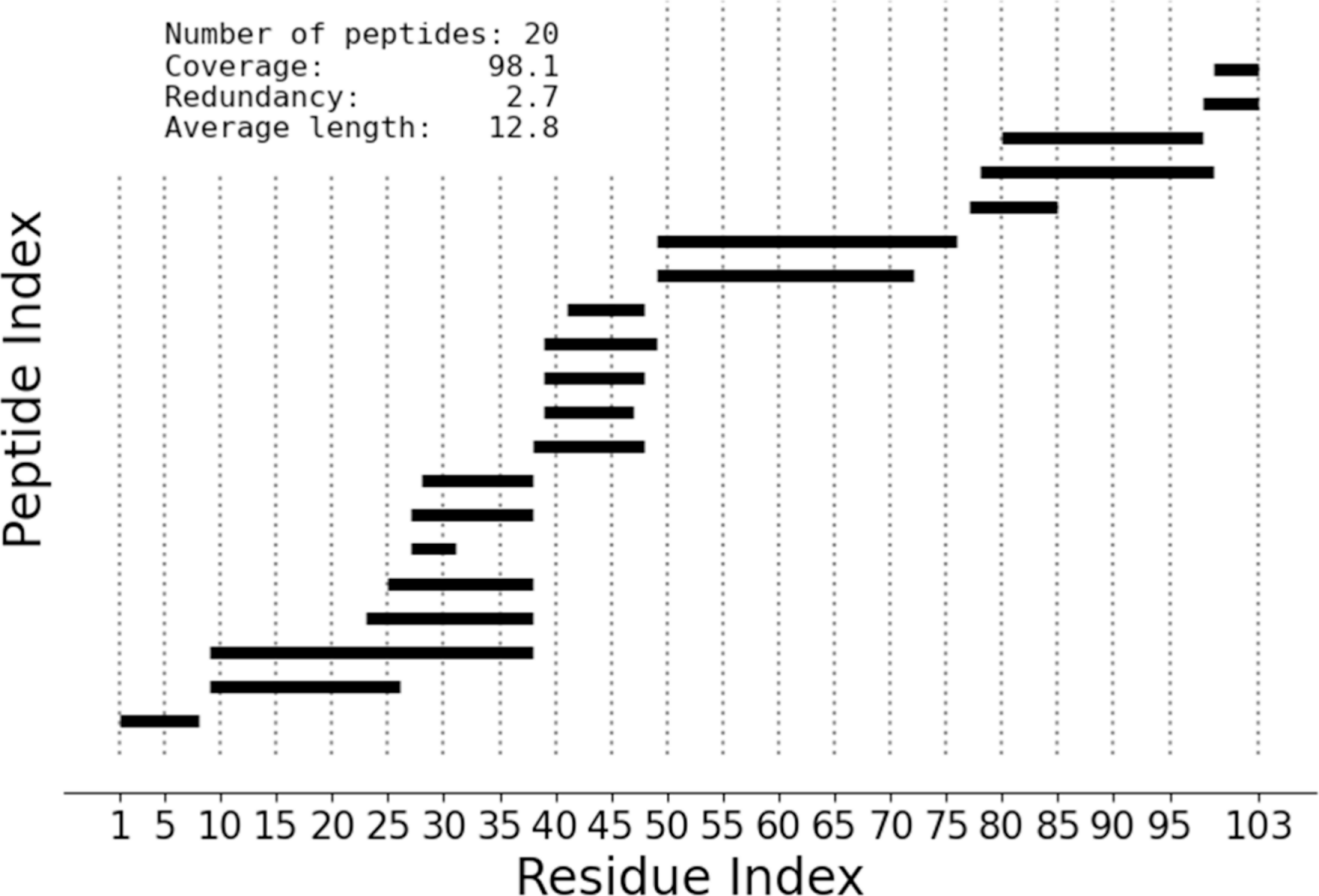
Example of a typical coverage map. Horizontal bars represent proteolytic peptides localized along the sequence of the protein. Number of peptides, sequence coverage, redundancy, and average length (number of amino acids) are reported.

The peptide list is generally reported in a coverage map (Figure 2), where peptides are depicted as horizontal bars and visualized across the sequence of the protein. The quality of the data set can be quantified mainly with 3 parameters: the number of peptides, the sequence coverage (the percentage of residues of the protein covered by the proteolytic peptides) and the redundancy (the “overlap”, defined as average number of proteolytic peptides available per covered residue).

### 3.1 Absolute and Fractional Uptake

**Figure 3:**
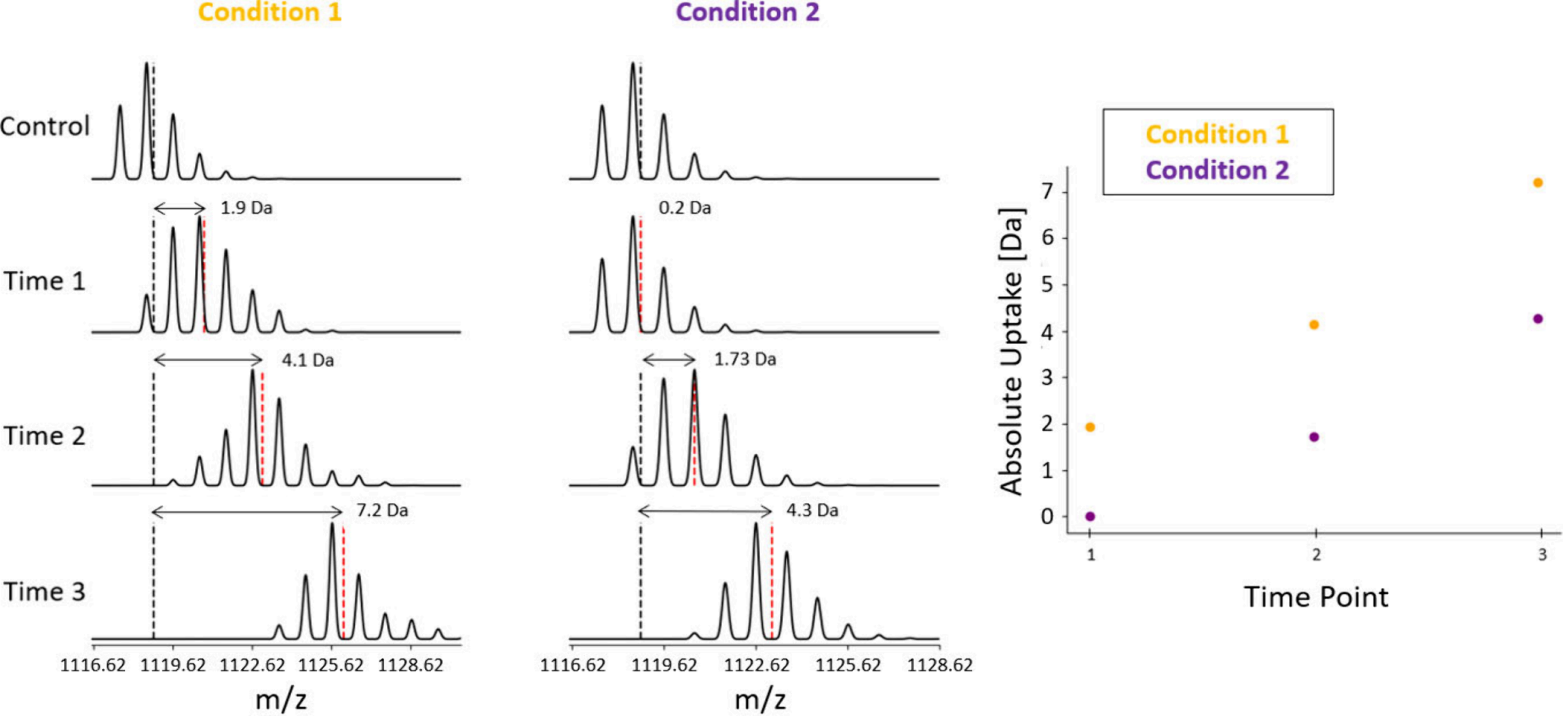
Isotopic envelopes of proteolytic peptides for different experimental conditions under analysis (simulated data). Data shown for visualization purposes only for one peptide under two arbitrary experimental conditions: condition 1 (orange) and condition 2 (purple). At increasing labeling times, the isotopic envelope shifts toward higher values of *m*/*z*. The centroid of the isotopic envelope, i.e., the intensity-weighted average, is monitored. The absolute uptake (right) is defined as the difference between the centroid of the envelope at a specific time and the centroid of the control (fully protonated) envelope. The absolute uptake of different conditions is compared.

After the generation of a peptide list and the manual or automated assignment of isotopic envelopes at different labeling times, the intensity-weighted average *m*/*z* of the isotopic envelope of the peptide is recorded as a function of time (Figure 3).

The measured *m*/*z* value (*m*_*z*_) at a specific charge state *z* is converted into a mass scale (*m*) using the following formula:
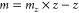
4

The mass increase (or absolute uptake) is defined as the difference of the mass of the peptide at labeling time *t* (*m*(*t*)) and the mass of the peptide in the control sample (*m*_0_):
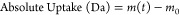
5

If the mass of the fully deuterated peptide (*m*_FD_) is measured,^68^ then the absolute uptake is commonly converted to the fractional deuterium uptake (*D*_Frac_):
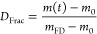
6

The measured fully deuterated sample often does not match the theoretical fully deuterated mass, which corresponds to the number of exchangeable amides (i.e., excluding prolines and the first/second residues^56^). This discrepancy arises because back-exchange (i.e., deuterium loss) can happen at different stages along the experimental workflow (section 3.2). When a fully deuterated sample is available, the fractional uptake in eq 6 represents the conventional back-exchange correction.

### 3.2 Back-Exchange

The Linderstrøm-Lang model (eq 1) considers “forward” HDX (i.e., H to D) to be an irreversible process, which is true only during the labeling phase (before quenching), when the protonated protein is exchanging within a 100% deuterated buffer, and when further processing steps from the quench onward are neglected. This is not the case in typical HDX-MS experiments, where the protein is diluted resulting in an 80–95% deuterated buffer. While higher dilution factors could reduce reverse exchange and more closely align with the theoretical model, they are often impractical because the resulting protein concentrations might fall below the detection limit of the mass spectrometer. During the HDX-MS experimental workflow, there are several steps at which *back-exchange*, i.e. partial loss of deuterium label, can occur (Figure 1). The deuterium labeling is performed in a highly (yet not purely) deuterated buffer (80–95%), and therefore *reverse exchange* (D to H, deuterium/hydrogen exchange) is occurring at the native protein level (e.g., with a 5% probability in a 95% deuterated buffer). From the quench onward, the deuterated solution is mixed with a water-based quench buffer (generally at a 1:1 ratio): forward exchange and back-exchange are competing mechanisms occurring at the protein level from quench to digestion and at the peptide level afterward. Additional back-exchange can occur during ionization and in the gas phase before detection in the mass spectrometer. To minimize back-exchange after the labeling phase, the temperature of the solution should be decreased (even below 0 °C) by placing the reversed-phase column for peptide separation in a refrigerated unit; but the digestion unit is usually kept at higher temperature to ensure efficient digestion.^69^ However minimized, it is not possible to completely remove back-exchange from the HDX-MS workflow, and therefore a proper quantification of back-exchange levels and consequent data normalization are highly desirable, but currently still lacking.

Most differential studies (i.e., where two or more experimental conditions of the protein are compared) do not perform any back-exchange correction and instead compare the absolute uptake (eq 5) of the same proteolytic peptides derived from different biological states of the protein, under the same experimental (technical) conditions. This procedure is correct only under the assumption that the extent of back-exchange is the same in the two experimental conditions, such that the denatured intact protein (from quench to digestion) and the peptides (from digestion onward) are fully unstructured or retain similar residual structure. The validity of this assumption is not straightforward: for example, Sheff et al.^70^ have shown that proteolytic peptides can retain residual structure in the LC column, hence different protein conformations may induce different back-exchange levels. Beyond differential studies, a proper back-exchange correction is essential if absolute and quantitative biophysical properties are required (such as exchange rates or protection factors, see section 4.4).

When implemented, the *standard* approach to correct for back-exchange is the acquisition of a fully deuterated sample and the normalization of absolute uptake values into fractional uptake (eq 6). There exist different protocols to acquire fully deuterated samples. For example, a fully deuterated control can be acquired by leaving the protein to deuterate for a time that is *long enough* to see the plateau in the kinetic uptake curves (e.g., for 12 h). In many settings, researchers decide to avoid this strategy because many proteins are unstable for such long times (their partial denaturation resulted in lower intensities in the chromatogram, or their aggregation causes false protection), and in rare cases membrane proteins retain regions so protected that their exchange after 12 h is negligible. A second strategy to acquire a fully deuterated control consists of diluting the protein in a deuterated buffer containing high levels of denaturant (e.g., 4 M urea) and leaving it overnight to exchange. A third strategy consists in performing offline digestion and deuteration of the proteolytic peptides. In the absence of a published study which systematically compares the results of the different strategies to acquire a fully deuterated sample, we recommend either of the latter two approaches.

**Figure 4:**
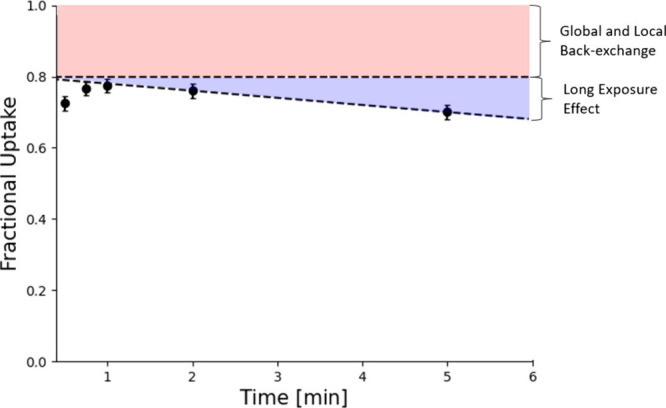
Back-exchange correction applied by the software DECA.^32^ The global and local back-exchange correction (red) produces a peptide-dependent plateau, resulting from both protein-dependent back-exchange (occurring from sample dilution into labeling buffer to digestion) and peptide-dependent back-exchange (occurring from digestion to detection in the mass spectrometer). The long exposure effect is an apparent back-exchange correction suggested by DECA which consists of a linear correction that is universally applied to all peptides.

As an alternative to the *standard* approach, the software DECA^32^ was implemented around the need for developing a back-exchange correction. The authors identified two distinct forms of back-exchange that can influence deuteration (Figure 4): they called these “global back-exchange” that occurs at the level of the intact, but denatured protein (from quench to digestion on the pepsin column), and “local back-exchange” that acts at the level of the peptide (from digestion to the point of injection into the mass spectrometer).^71^ Back-exchange causes the deuterium uptake curve to plateau at a value lower than the theoretical fully deuterated mass.

The authors of DECA^32^ also identify a “long exposure effect”, which causes later time points to slowly deviate from the fully deuterated plateau. There are a number of possible reasons which can cause such effects, the most obvious being related to the stability of the protein at longer time points where aggregation could lead to apparent protection against exchange. In addition, ambient moisture can also lead to deuterium loss in the sample causing a drop from the deuterated plateau. During a multiday series of time points and replicates, protein samples may end up being kept at 0 °C for several days in the autosampler; but issues arising from this can be addressed by careful experimental design (e.g., mixing replicates of different time points randomly, or regularly replacing the protein sample with fresh aliquots). Protein stability tests done prior to HDX analysis are also helpful. In addition, the DECA paper describes an experimental artifact which can be misinterpreted as an additional form of back-exchange, caused by different liquid handling procedures at short and long time points. For example, when a LEAP robot is used for time points below 2 min, the mixing syringes skip a step, and this results in a slightly lower back-exchange. DECA allows to correct for global and local back-exchange by the application of a scaling factor, as well as accounting for this long exposure effect by the application of a universal, linear correction to all peptides. A recent paper by Wrigley et al.^72^ expanded on the subject, confirming that automated liquid handling procedures can indeed introduce a large variability to the measured deuteration. While liquid handlers provide excellent efficiency with respect to manual pipetting, the number of steps involving syringe operations with small liquid volumes that occur during an HDX-MS experiment can be source for volumetric errors, which can cause minor differences in the final deuterium concentration or in pH, and in turn can be sufficient to cause significant differences in the uptake curves of peptides. These robot-related issues can be resolved by tracking the performances of the liquid handler over the different operations performed during the workflow and consequently optimizing the robot methods (e.g., changing the needle position or depth) to reduce the variability in the measured deuteration.

The corrections for back-exchange mentioned in this section underscore our limited knowledge of the phenomenon, and several questions remain unsolved: what is the best strategy to acquire a fully deuterated sample? What percentage of forward- and back-exchange is occurring during deuterium labeling, during the quench procedure, during digestion and in the gas phase, respectively? Can we reduce or eliminate any of these contributions, or at least control them so that they can be quantified accurately? Fundamental studies are needed to systematically answer these questions, for example studying the behavior of model proteins while varying the deuterium percentages in the quench buffer, or by replacing the water-based LC solutions with deuterium-based equivalents. While a proper back-exchange correction offers minor advantages for differential studies, it becomes crucial when integrating experimental data with modeling (i.e., for the methods described in section 4.4) as the *standard* back-exchange correction may yield inaccuracies in that it assumes all residues in a peptide back-exchange to the same extent.

### 3.3 Replicates

The reported mass increase (eq 5) or fractional uptake (eq 6) is averaged over the available number of replicates, generally limited to 3 or 4. The main factor limiting the number of replicates in HDX-MS experiments is the cost associated with additional sample consumption and instrument runs. The error associated with the experimental measure is either the standard deviation or the standard error (standard deviation divided by the square root of the number of replicates). Replication allows to assess whether the observed differences are likely to occur by chance or not,^73^ and to ensure the reliability of the conclusions drawn from the observed data. Increasing the number of replicates results in a more precise inference regarding differences between groups.^74^

Not all replicates are equivalent. In the context of HDX-MS experiments (as well as proteomics and other biophysical techniques), replicates can be divided into two categories:^75^*biological* replicates, which can derive from (i) independent protein expression or isolation from source tissue and (ii) steps prior to the addition of deuterium (e.g., incubation with a ligand or membrane), and *technical* replicates, which can be in turn subdivided into three subcategories: (i) *labeling* replicates, corresponding to independent deuterium additions to the same protein stock material, testing sample conditions during labeling (timing, pH, temperature) and LC-MS parameters, (ii) *analysis* replicates, which are repeated LC-MS injections of the identically labeled sample, testing the variables from the point of injection into the LC-MS system, and (iii) *processing* replicates, that are software-based replicates on the same set of data that test the computational parameters and data processing reliability.

These types of replicates have a well-defined hierarchy: inferences drawn from biological replicates are more powerful than inferences made from technical replicates.^76^ Currently available software are not able to account for these differences. Multiple approaches are being used to account for technical and biological replicates. A commonly applied approach is averaging the deuteration values of the technical replicates within each biological replicate. This strategy yields consistent estimates, but gives incorrect uncertainty estimates, leading in a differential study to a higher number of false findings. Another approach is to analyze each biological replicate separately. Such an approach ignores the dependencies of the technical replicates within a biological replicate and ignores relevant biological variation, limiting generalization and replication of results. Both approaches should be avoided as they do not properly acknowledge the data structure. A third approach is the use of statistical models and tests that account for the level of replication (technical/biological), such as linear mixed models, which are described in more detail in section 4.2.3. It is worth mentioning that most published studies report only technical replicates. As a general recommendation, biological replicates should be prioritized over technical replicates whenever possible. When the number of biological replicates is limited (e.g., when there are only two), collecting data from both biological replicates, along with multiple technical replicates, allows for more robust inferences than relying on technical replicates alone. In the latter case, implementing a mixed effects model is essential to appropriately account for the level of replication.

### 3.4 Charge State Effect

Many peptides can be found in electrospray ionization mass spectra with more than one charge state, and the apparent deuterium uptake behavior of the same peptide at different charge states can show systematic differences. This is a well-known but rarely reported effect,^77^ and is caused by back-exchange postionization in the electrospray source and gas phase of the mass spectrometer. Guttman et al.^78^ demonstrated that this charge state offset, which occurs to different extents on different instruments, is due to nonuniform gas-phase exchange with water vapor within the ion optics of the instrument. For example, such back-exchange in a Waters Synapt G2-Si can be reduced (yet not completely removed) by adjusting the settings of the StepWave ion guide (mainly DC offset potential and the traveling wave height and velocity). There are two policies implemented by the available software packages: (1) the mass increase (or fractional uptake) is reported as an average over the available charge states of a peptide; (2) only the mass increase (or fractional uptake) of the most intense charge state is reported. We note here that neither option is ideal as the first one is not able to account for the possible systematic difference in deuterium loss between charge states, and the second introduces a *selection bias* in the analysis. A third alternative, which probably represents the best option, is to analyze different charge states individually and check that the results are consistent across the different charge states; in this latter strategy replication may be a problem as not all charge states are found for each replicate or condition.

### 3.5 Linderstrøm-Lang Model for Peptide-Level Data

The Linderstrøm-Lang model (eq 1) describes HDX at the level of the single amino acid. However, most HDX-MS experiments detect the deuterium uptake of a protein via its proteolytic peptides. For this reason, HDX-MS data are *coarse-grained*: they monitor the behavior of entities (peptides) that are smaller than the whole system (protein) but bigger than the smallest resolvable unit (amino acid).

For a peptide with N exchangeable residues (i.e., excluding prolines), the deuterium uptake (D) of the proteolytic peptide can be written, using the Linderstrøm-Lang model, as the sum of the uptake *d*_*i*_ of its residues:
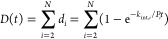
7

The first amino acid (*i* = 1) is excluded from the contributing residues because its amide hydrogen is lost upon digestion. Sometimes, depending on the sequence of the proteolytic peptide, also the second residue should be excluded, assuming it rapidly back-exchanges during the quench step and the deuteration is lost.^79^ In certain sequences, such as those containing histidines, the back-exchange rate for even a middle amide can be so fast that all deuterium will be nearly lost by the time the peptide is analyzed, and therefore the amide will not contribute to the overall deuterium measurement. A paper by Hamuro nicely summarized the expected deuterium loss for different sequence contexts.^56^

One of the challenges for the analysis of HDX-MS data is to retrieve single residue information (i.e., the individual protection factors) from peptide-level data. In statistics, this problem is defined as *underdetermined*: the number of parameters to be estimated is greater than the number of experimental data points.^38^ In the case of an isolated peptide formed by *N* residues, whose exchange has been monitored at *K* time points, we can distinguish two scenarios (assuming for simplicity that experimental error is negligible): (i) when *N* > *K* (which is the most common case as the average peptide length is ∼10 amino acids and the HDX is generally detected at between 3 to 5 time points), the consequence of under-determination is that there are multiple solutions (i.e., patterns of protection factors) in agreement with experimental data; (ii) when *N* < *K*, there is one only solution in agreement with experimental data, but the extracted protection factors cannot be assigned to a specific residue (indeed, eq 7 does not account for the order of the residues). Using the complementary information contained in overlapping peptides helps reducing the multiplicity of the solutions, up to the point that single residue information can be in principle obtained in an *ideal* data set where all peptides differ by one amino acid only.^35^ This is usually not the case, and other approaches have been used instead. Different methods aiming to extract protection factors from HDX-MS data are discussed in detail in section 4.4.

### 3.6 Visualization of Preprocessed Data for One Condition

**Figure 5:**
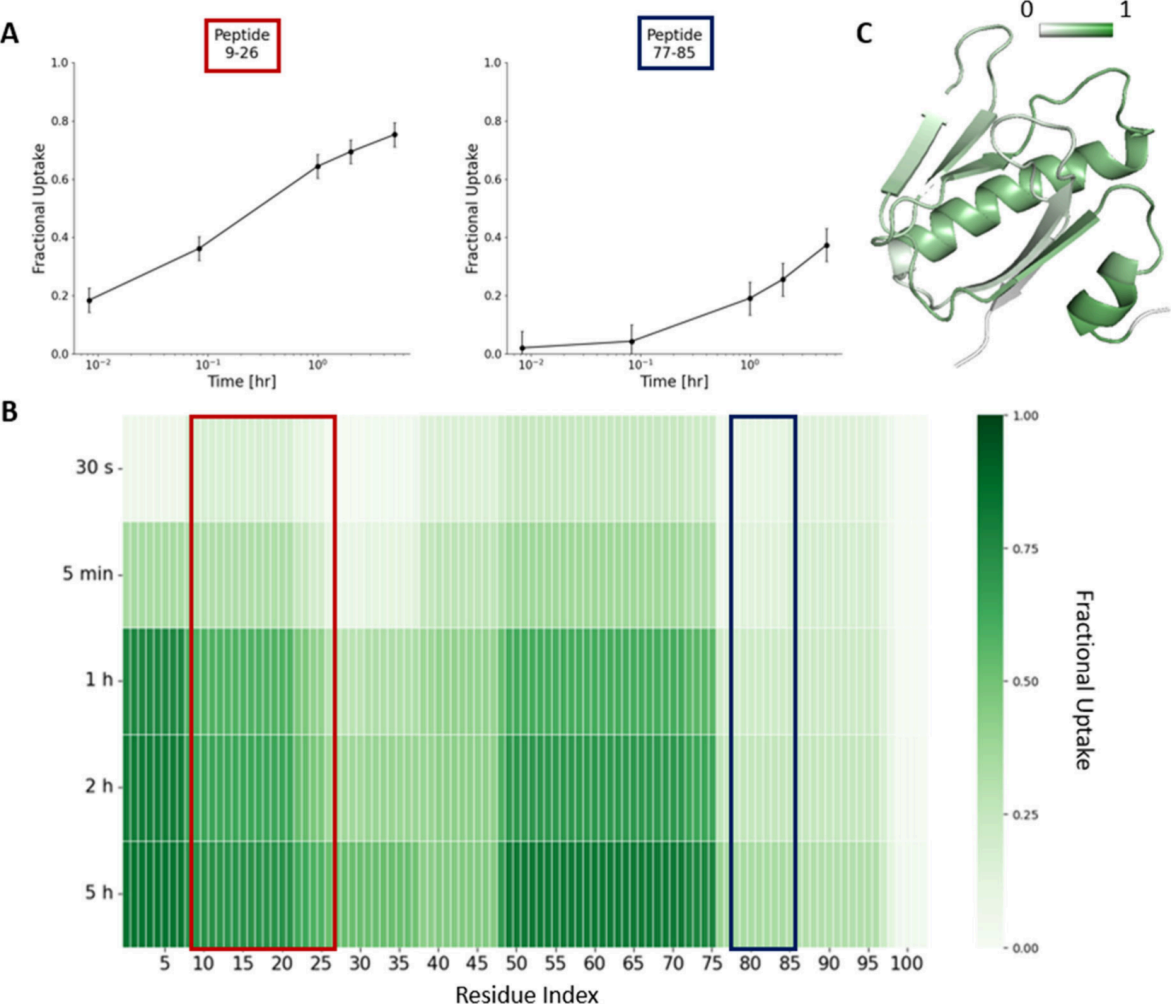
Visualization of preprocessed data for one condition. (A) The uptake curve shoes the fractional uptake as a function of the labeling time. Average and standard deviation are displayed. (B) The heat map shows the fractional uptake as a function of the labeling time along the sequence of the protein. The fractional uptake of each residue is the equal fraction of the average fractional uptake of the proteolytic peptides covering that specific amino acid position. (C) The heat map is projected onto the protein structure at labeling time 1 h.

Preprocessed data are generally visualized through *uptake curves* (Figure 5A), reporting either the (average) mass increase (eq 5, in Da) or the (average) fractional uptake (eq 6, in % of the maximum) at different labeling times of proteolytic peptides. Heat maps (Figure 5B) can be generated to visualize the time evolution of the uptake at a pseudoresidue resolution along the sequence of the protein. Generally, the deuterium uptake of a residue at a specific labeling time is calculated as an equal fraction of the average over the mass increase (or fractional uptake) of the peptides covering the amino acid position. However, this calculation varies from software to software. For example, DECA^32^ generates heat maps by assigning residues to the most representative peptide available (i.e., the shortest). An alternative approach uses weighted averaging, where peptide uptake values are weighted by the inverse of the peptide’s length.^80^ If the structure of the protein is available, the pseudoresidue uptake provided by the heat map can be mapped onto a 3D structure (Figure 5C). Many pieces of software provide a PyMol script to generate these plots. HDX-Viewer^81^ is an online tool that was developed to provide an easy-to-use interface to visualize deuteration within the structure of the protein. It is worth stressing that these representations are useful tools to map experimental data onto protein models, but they can be misleading as the high resolution achieved is artificial.

Alternative visualization tools involving multivariate analysis, such as principal component analysis (PCA) or spectral mixture analysis (SMA), can be used to check the quality of the data (e.g., to see if samples from the same condition or time group together),^31^ and have been proven useful to show whether compounds with similar in vivo properties were forming statistically distinct clusters.^82^

### 3.7 Preprocessing Data with Commercial Software

It is needless to say that a correct preprocessing of data is a crucial step in the data analysis workflow of HDX-MS experiments as poorly curated data can lead to incorrect biological conclusions. In the context of HDX-MS data, we define as data “pre-processing” the steps of the data analysis workflow that start from the raw data and a list of potential peptides, and lead to the generation of deuterium uptake curves. Most HDX-MS publications utilize commercial software to preprocess raw data, namely DynamX for Waters instruments and HDExaminer for Thermo Scientific/Trajan. These two programs share common features: they require knowledge of the protein sequence, in the form of a peptide list (whose generation is later discussed in section 4.1), and the raw HDX-MS data as inputs. They enable the identification and assignment of the undeuterated and labeled isotopic envelopes, to calculate the absolute uptake of peptides, to visualize the data through coverage maps, uptake plots and heat maps; and they return a spreadsheet containing the information about the uptake of peptides over time. They mostly differ on how the user can interact with the isotopic envelopes identified by the software and edit them manually. They also share the same limitations: they do not perform statistical analysis, back-exchange correction or fully deuterated normalization, which must be done using third-party software packages. While these commercial software packages are critical for preprocessing raw data, they must be integrated with other software packages to achieve comprehensive and publishable results.

Two additional commercial software packages, Protein Metrics (Dotmatics) and HDXWorkbench,^27^ must be mentioned. They both aim to integrate the entire analysis workflow into a single platform. These tools facilitate automated peptide search starting from the raw data (section 4.1), identification and assignment of undeuterated and labeled isotopic envelopes, and provide statistical methods for differential analysis (section 4.2). Additionally, they offer features for multimodal analysis (section 4.3) and enhanced spatial resolution (section 4.4).

## 4 Computational Tools for HDX-MS Data Analysis

### 4.1 Automated Peptide Search and Identification

At the beginning of the data analysis pipeline, it is necessary to identify peptides and assign peaks in the raw mass spectra. As already mentioned, the identification of proteolytic peptides is generally performed using commercial software included with the instrument, and the quality of the isotopic envelopes of each peptide is then checked manually. Peptides showing saturation (the intensity of the peptide signal exceeds the instrument’s dynamic range, altering the shape of the isotopic envelope), multimodal behavior which can be due to EX1 or EXX kinetics (see section 4.3), carryover (peptides retained on the fluidics system from the previous sample injection) or ambiguous assignment (e.g., due to the presence of different envelopes in the same *m*/*z* range) can be kept or rejected depending on the practitioner. There are no clear guidelines on how to perform these assignments, and policies vary from group to group. Consequently, this preprocessing step is time-consuming and user dependent. Moreover, a major disadvantage of commercial software packages is that they do not allow to export the isotopic envelopes but only the average *m*/*z* values, making it hard to retrieve information about the detailed characteristics of the assigned mass spectra of the peptides.

Tools have been developed to tackle the drawbacks mentioned above. ExMS2^36^ proposes an automated peptide validation pipeline to speed up the peptide quality checks. This requires as input a peptide list generated by SEQUEST/Bioworks (alternatively Proteome Discoverer or MassLynx) from the control sample (undeuterated, all-H protein). Each peptide is associated with its chromatographic retention time (RT) and its *m*/*z* value. For each peptide in the list, ExMS2 selects the MS scans within the known RT window and compares the experimental spectra with calculable mass spectrometric information, such as monoisotopic mass, charge state, and isotopic peak positions. The process is repeated for each sample at the different time points available. ExMS2 records *m*/*z* values and relative intensities for each isotopic peak to define the shape of the isotopic envelope. The recorded peptides are validated through 12 quality tests (six performed on a peptide level and six on a multipeptide level), for example checking if the overall peak intensity is above a certain threshold or if the peak is within the possible *m*/*z* range for a peptide in the list. Peptides failing one or more tests are flagged and can be manually inspected.

Mass Spec Studio^47−49^ first proposed HXpipe (peptide identification and peptide evaluation) as a tool for automated peptide search and validation. Two searches are performed independently: (i) an MS/MS search, which looks for peptides in the LC-MS/MS files using one of two available search engines (MS-GF+ or OMSSA+); (ii) an MS search, which uses a peak picker that scans within the LC-MS/MS data to create a library of chromatographic features, which are then compared with theoretical isotopic distributions calculated using Senko’s Averagine model^83^ for peptides. The results from the two searches are then combined together. A new module, named AutoHX,^50^ has been implemented into Mass Spec Studio, to facilitate (and ideally remove) the manual inspection of the peptide search. AutoHX leverages the information contained in the deuterated fragment peptides to (i) validate the identity of the peptide and (ii) confirm the deuteration level of the precursor peptide by checking that the deuterium content of the peptide fragments has a linear relationship with the fragment length. This automatic authentication and validation, which exploits MS/MS data and uses deuterium-scrambled CID or HCD fragments as surrogates that confirm the identity and the deuteration value of any given peptide, yields objective results with known certainty, rather than biased results with unknown certainty provided by a traditional approach, which uses MS data only and is followed by laborious manual (i.e., user dependent) validation.

In the previous paragraphs, we reviewed different software packages designed for automated peptide search and identification from raw LC-MS data. These tools bridge the gap between researchers and raw data, facilitating the preprocessing and validating peptide-peak assignments along with deuteration values at specific labeling time points. While they serve as a viable alternative to the commercial software described in section 3.7, their adoption is limited mostly due to a lack of know-how outside of the group of researchers which generated them. Although their documentation is generally robust, we believe that additional tutorials and workshops for HDX users would help with their broader adoption in the community, and this has also been suggested at the recent conference of the International Society of HDX-MS in April 2024 in Monterey, CA/USA. Among the software reviewed, Mass Spec Studio stands out as the most comprehensive; integrating automated peptide search and identification with subsequent workflow steps, such as differential analysis (section 4.2) and multimodal analysis (section 4.3). It is crucial for software developers to consider the integration of diverse data types (e.g., tools for analyzing ExD or UVPD fragment data) and ensure easy access to processed data (e.g., straightforward export of processed isotopic envelopes). Likewise, instrument manufacturers should be encouraged to enable the export of HDX-MS data sets with key information such as the isotope patterns and charge states of peptides.

### 4.2 Differential Analysis

The analysis of HDX-MS data generally relies on a side-by-side comparison of two (or more) conditions (e.g., a protein in absence or presence of a ligand). For each proteolytic peptide, the difference in deuterium content obtained from the different experimental conditions is classified as *significant*, or not, using thresholding and/or statistical tests and models. Differences in the uptake pattern of peptides highlight regions of the protein where a structural perturbation has occurred (binding site, allosteric change, etc.). There are two strategies to analyze differential HDX-MS data. The first (and most used) looks at the difference in deuterium content *at a given time point*: manual thresholding (section 4.2.1), simple hypothesis tests (section 4.2.2), or linear models (section 4.2.3). The second approach compares deuterium uptake curves (section 4.2.5). These two strategies, summarized in Table 2, are described in this section.

**Table 2:** List of Software to Analysis Differential HDX-MS Data

differential analysis	
name	approach
DECA^32^	*t* test
Deuteros 2.0^34^	linear model
HaDeX^39^	*t* test
HD-eXplosion^40^	*t* test
HDflex^42^	*t* test
HDXAnalyzer^43^	linear model
Hdxstats^45^	functional analysis
Mass Spec Studio^47−49^	*t* test
MEMHDX^51^	mixed model

#### 4.2.1 Manual Thresholding

One approach used to analyze differential HDX-MS data consists of defining a manual threshold for the difference in deuterium content between conditions.^84−86^ This threshold is set to a predefined value (generally 0.5 Da^87^) or based on the standard deviation of the data (e.g., using the pooled standard deviation^88^). If the difference in deuterium content at a specific time point exceeds this threshold, then the peptide is classified as *different.* This approach ignores the variability of the peptide-deuteration levels and can therefore lead to false findings.^31,88^ For example, defining a strict threshold to reduce the number of false positives leads to ignoring small yet biologically relevant differences in deuteration (i.e., false negatives), while a generous threshold limits the number of false negatives, but results in many false positives. We therefore advise against manual thresholding approaches and advocate the use of statistical methods to test for differences, as they account for the variability of measured deuterium levels and thus control the number of false findings.

#### 4.2.2 Simple Hypothesis Testing

In simple hypothesis testing, a null hypothesis (*H*_0_) is compared against an alternative hypothesis (*H*_a_). In differential HDX-MS, the null hypothesis commonly states that there is no difference in the deuterium content of a peptide between two or more conditions, while the alternative hypothesis claims that there is a difference. Statistical tests are used to test the null hypothesis, i.e., to reject or not to reject the null hypothesis, by calculating a test statistic. Student’s *t*-test is commonly used when one wants to compare the means between conditions/groups. Student’s *t*-test is in essence a signal-to-noise ratio test, where the difference in the average deuterium content is divided by a nuisance parameter, which is a function of the variability of the data. The larger this ratio, the more likely the null hypothesis can be rejected in favor of the alternative hypothesis. The exact value (*critical value*) required to reject the null hypothesis depends on the number of observations and the specified significance level (α). Generally, a *p*-value is reported instead of the critical value. If this *p*-value is smaller or equal than α, then the null hypothesis can be rejected. When more than two conditions have to be compared, an *F*-test which tests if at least one mean is different from the others. Student’s *t*-test and the *F*-test both assume that the data is normally distributed. If this is not the case, nonparametric alternatives, i.e. the Wilcoxon signed-rank test or Mann–Whitney *U*-test and the Kruskal–Wallis test can be used. Note that when the underlying assumptions of the parametric tests are true, the nonparametric test statistics are less powerful than their parametric counterparts, i.e. they identify less differences in deuteration that are truly different as statistically significant.

Differential HDX-MS experiments are generally done with a limited number of replicates. As a consequence, the variability of the deuterium content of a peptide is harder to estimate accurately. This can potentially lead to more false findings, i.e., more false positives and/or false negatives. Claesen et al.^31^ proposed using moderated *t*- and *F*-statistics instead of Student’s *t*-test and *F*-statistics. These test statistics borrow information from other peptides with similar deuteration values to reliably estimate the standard error of the mean, resulting in a lower number of false findings.

#### 4.2.3 Linear Regression Models

Although simple hypothesis testing is a convenient way to test for differential hydrogen–deuterium exchange (per peptide), uniting all hypothesis tests in a linear regression model allows to directly estimate differences between the different groups or conditions (protein states). Additionally, it allows to correct for other factors (*confounders*) that could have an effect on the deuteration.

In a linear regression model, the response variable or dependent variable (**y**) is a linear function of one or more explanatory or independent variables (**x**):
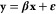
8where ε is the residual error and follows a normal distribution (**ε** ∼ *N*(μ = 0,σ^2^)), and **β** are regression coefficients that are derived from the data and express the effect of the explanatory variables. The reader would be familiar with the simplest case of a straight line with slope m and intercept *q*: *y* = *mx* + *q*, which is equivalent to eq 8 in the case  and . In the latter case, a linear regression model fits a line to the data and allows to evaluate the effect (*m*) of the explanatory variable *x* on the response variable (*y*). The same linear regression model in eq 8 to compare means of different groups and check whether they are significant different from each other by testing the estimates for **β** with a *t* test. The advantage of a linear model over a *t* test is that it can account for more than one explanatory variable at a time.

Deuteros 2.0^34^ and HDX-Analyzer^43^ implemented the following multiple regression-model, where the absolute deuteration of a peptide **D** is modeled as a function of the explanatory variables **Time** (labeling time) and **State** (biological state of the protein):
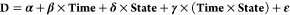
9where **α** represent the intercepts of the model, **β** the regression coefficients for the labeling time points, **δ** the regression coefficients for the different conditions/states, **γ** the regression coefficients for the interaction of state and time, and **ε** the residual errors of the model. In this model, **Time** and **State** are categorical variables, i.e., characteristics that are not quantifiable. In other words, if we have three time points, then they are treated as time point number one, two and three (rather than, for example, 30 s, 5 min and 1 h)—as a consequence, interchanging the time points would not affect the results. This regression model can be used to test whether changes in the deuterium-uptake of a peptide are associated to changes in state and/or time, and the interaction between state and time. Note that including the labeling time points as a continuous variable (rather than categorical variables) is possible, but time would have to be transformed to account for the nonlinear relationship between labeling time and deuteration uptake, or a nonlinear regression model would be required. The proposed multiple regression model can also be extended by adding other (categorical) variables, for example, charge state.

Depending on the experimental design, HDX-MS data can have correlated and/or repeated measures, for example, when an experiment is run in different batches or when both technical and biological replicates are acquired (see section 3.3). In the latter case, for example, we expect data from within the same biological replicate to be more similar to data between different biological replicates. The linear model, as defined in (eq 8 and 9), can be updated to a linear mixed effects model to account for the correlation present in the data:
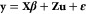
10where **ε** represent the residual error of the model and follows a normal distribution (**ε** ∼ *N*(0,σ_ε_^2^)), **u** is an unknown vector of random effects and also follows a normal distribution (***u*** ∼ *N*(0,σ_*u*_^2^), and **Z** is a design matrix for the random effects. The random effects, **u**, account for the correlation that is present in the data.

To clarify the content of eq 10, we now provide two examples where using a mixed model is advisible in the context of HDX-MS experiments.

Suppose we performed an experiment with 3 biological replicates, and 3 technical replicates per biological replicate (i.e., 9 experiments). The design matrix **Z** in eq 10 indicates which observations come from which biological replicate. (For a peptide *i* at time point *j*, we can assign ***uZ*_*ij*_** = 1 for all technical replicates of the first biological replicate and similarly ***uZ*_*ij*_** = 2 and ***uZ*_*ij*_** = 3 for the technical replicates acquired from the second and third biological replicate).

**Figure 6:**
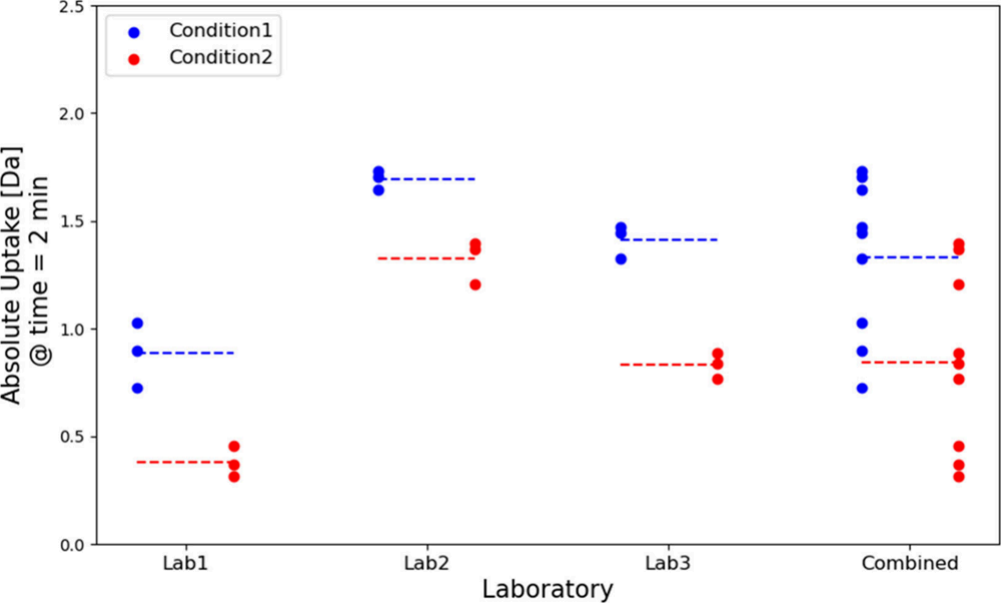
Example of random effects affecting the outcomes of an analysis. The absolute uptake of a proteolytic peptide of a protein has been measured at labeling time 2 min under two different experimental conditions (Condition 1, blue; Condition 2, red) in three different laboratories. Each laboratory classifies the peptide as significant (*p*-values are 0.0066 for Lab1, 0.0048 for Lab2, 0.0005 for Lab3). When the results are combined together into a single data set, the peptide is no longer significant (*p*-value = 0.0183).

Alternatively, suppose that the same protein was studied under different experimental conditions in three different laboratories, and we wanted to combine all measurements into a single data set to perform a separate meta-analysis. The different protocols implemented by the different groups (for sample handling, automation of the LEAP robot, different parameters for the LC-MS gradient, etc.) introduce random fluctuations to the deuterium uptake value of the same peptide under the same experimental condition. Differences in uptake between conditions (for the same peptide at the same time point) are systematic and should be visible, but combining the results from the different laboratories without considering this as a source of random effects might introduce a bias into the outcomes of the experiment. For example, a peptide with significant differences correctly detected (i.e., a true positive) by the three different laboratories might be misclassified as nonsignificant if all measurements were combined (Figure 6). A mixed model can deconvolve the effect of the standard deviation of the different laboratories on the standard deviation of the combined data set.

MEMHDX^51^ is the only software that implements a mixed model for the analysis of HDX-MS data. Here, time and experimental condition represent fixed effects and the replicated or repeated measures are considered as a random effect, meaning that each technical replicate is assigned to a *different* random effect. The GUI version of the software only allows the user to perform a traditional differential analysis, and it does not allow the cross-experiment statistics described in the experiments above, which can however be performed using the multiple statistical packages in R, such as nlme.^89^

#### 4.2.4 Multiple Testing or Multiplicity

Hypothesis tests (such as the *t*-test) are prone to false positive results when multiple comparisons are performed simultaneously, i.e., comparing peptides across conditions at each time-point separately. In order to control the probability of finding false positives, several multiple testing or multiplicity correction approaches have been proposed that adjust the *p*-value.^90^ The best-known multiple testing correction method is the Bonferroni correction, which divides the significant threshold α by the number of comparisons *m*, therefore the adjusted significance threshold reads α* = α/*m*. However, the Bonferroni method is very conservative,^31^ i.e. it leads to a very high number of false negatives. Another well-known approach is the Benjamini-Hochberg procedure,^91^ which is less conservative than the Bonferroni approach. Hageman and Weis proposed a *hybrid* approach that combines *t*-tests with manual thresholding to correct for multiplicity:^88^ the difference in deuterium content between two conditions is classified as statistically significant if two conditions are met simultaneously: (i) the *p*-value returned by the *t*-test is smaller than the significance level (α) and (ii) the difference in deuteration is greater than a predefined threshold. This hybrid approach is implemented in HaDeX,^39^ HD-eXplosion^40^ and Mass Spec Studio.^47^

#### 4.2.5 Comparing Deuterium Uptake Curves

Crook et al. introduced a novel approach to the analysis of HDX-MS data in the framework of functional analysis.^45^ Experimental uptake curves of peptides are fitted with a Weibull model (also referred to as stretched exponential) of the form:
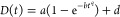
12where the parameter *d* represents the mass at time 0 (no exchange; undeuterated), which is inferred from the data; *a* controls the value at which the exchange reaches a plateau (maximum incorporation); *b*, the exchange rate constant, which models the exchange kinetics; *q* refers to additional factors that are deflecting the uptake curve from a single exponential behavior. The stretched exponential in eq 12 approximates the multiexponential behavior derived from the Linderstrøm-Lang model (eq 7).

The Weibull model (eq 12) is fitted with experimental data from two conditions and tools of functional analysis are implemented to assess whether the curves are significantly different. The underlying null hypothesis of functional analysis is that the same parameters can fit experimental curves from both conditions. The alternative hypothesis is that two independent models describe better the data. The *p*-values are returned by an F test, and multiple testing corrections (see section 4.2.4) can be applied (as for *t* tests, linear models and mixed models).

When using a linear model, time is modeled as a categorical variable: changing the order of time points does not affect the results of the analysis. With a mixed model, the random effect can account for the correlation present between time points for a given peptide. The major advantage of the functional model implemented is the possibility of explicitly modeling the deuterium content as a function of time, which allows to incorporate intrinsic exchange rates of the residues forming a peptide. This comes at the cost of acquiring a relatively large number of *informative* time points (early/late, spacing) to properly sample the uptake curve of each peptide.

#### 4.2.6 Visualization of Differential Analysis

**Figure 7:**
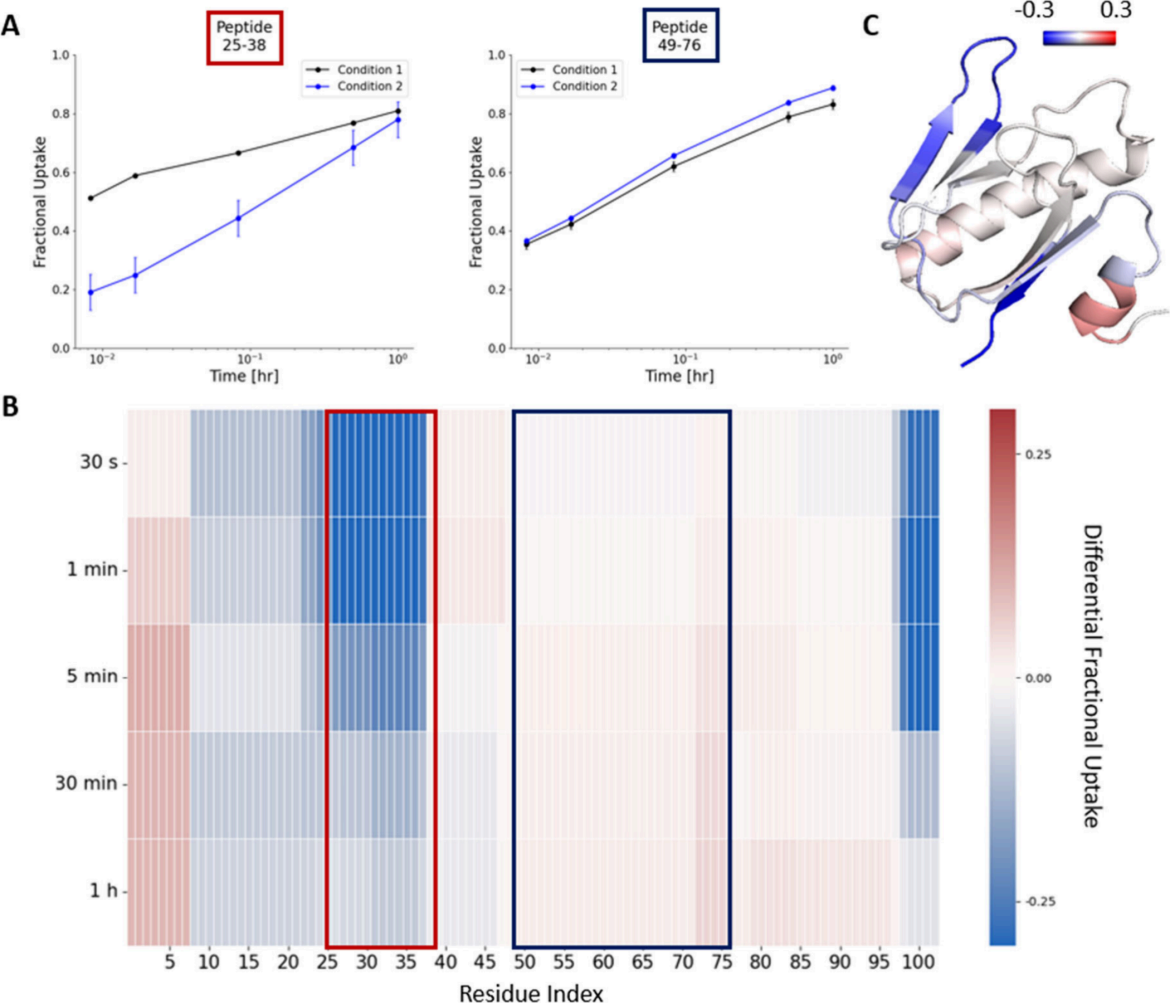
Qualitative visualization of HDX-MS differential analysis. (A) Uptake plots for peptides covering residues 25–38 and 49–76 are shown for two different experimental conditions. Differing curves highlight structural changes in this area of the protein. (B) Differential heat maps show the difference in uptake between two conditions as a function of the labeling time and along the sequence of the protein. Blue regions identify areas where Condition 2 is more protected than Condition 1; red regions correspond to areas where Condition 2 is less protected than Condition 1. (C) The differential heat map is mapped onto the 3D structure of the protein at labeling time 5 min.

The tools to visualize data for a single condition (uptake plots, heat maps and 3D structure visualization, see section 3.6) can also be used to qualitatively visualize the results of a differential analysis (Figure 7). Differential heat maps show the difference in uptake between 2 conditions rather than the mass increase of a single condition. These differences are often mapped onto a 3D protein structure, with a color scheme showing regions in white without significant differences, in blue those that are more protected in the target condition and in red regions that are less protected.

The plots in Figure 7 do not show the results provided by the statistical test used. The results of a differential analysis are generally reported in publications using Woods plots: proteolytic peptides are visualized across the sequence of the protein with horizontal bars and positioned along the *y*-axis according to the difference in uptake between two conditions; peptides showing statistically significant differences are highlighted (in blue or red). The statistical significance can be defined either by a single threshold on the *p*-value (*p*-value < α) or by a double threshold on the p-value and on the difference in uptake. If a *t* test, a linear model, or a mixed model is used, then each time point will be visualized on a different Woods plot; if functional analysis is implemented, then the results of the whole time-course will be displayed in a single Woods plot.

The volcano plot is an alternative tool to visualize the results of a differential analysis. Each proteolytic peptide is a point in the plot: the horizontal axis represents the difference in uptake between conditions; the vertical axis shows −log(*p*-value), which can be considered a measure of the statistical significance (the *p*-value depends on the statistical test implemented): the higher the differences between conditions, the lower the *p*-value, and therefore the higher −log(*p*-value). The volcano plot is ideal to visualize statistically significant peptides using the double threshold (on the *p*-value and on the difference in uptake), but it does not visualize the location of the peptide along the protein sequence.

**Figure 8:**
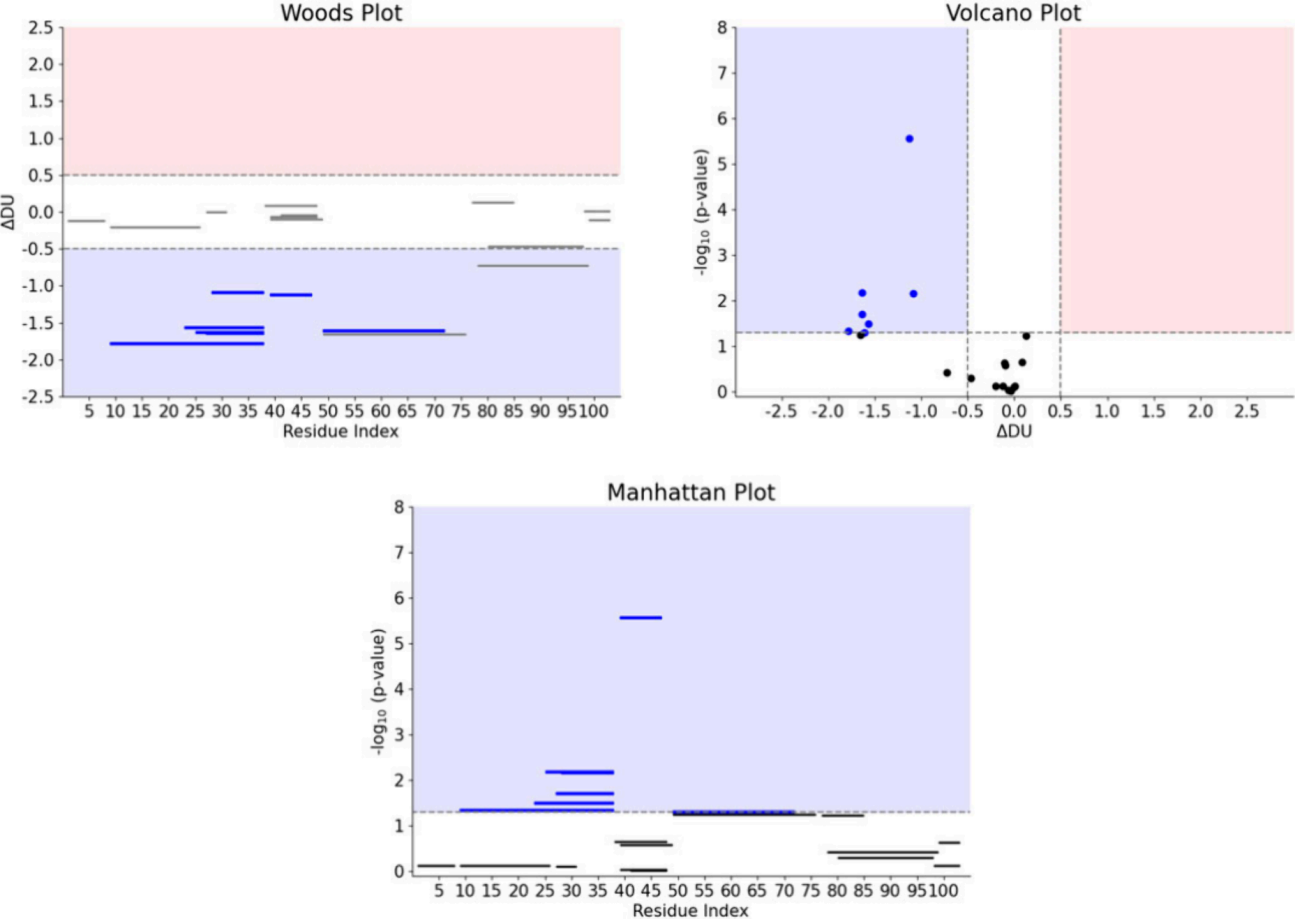
Quantitative visualization of the results of differential analysis on the same data set using Woods plots, Volcano plots, and Manhattan plots. See main text for plot description.

The Manhattan plot is another alternative tool to directly visualize the *p*-values returned by the chosen statistical test along the sequence of the protein. In this plot, the horizontal axis represents the peptide index, while the vertical axis shows the statistical significance (−log(*p*-value)). Alternatively, as shown in Figure 8, peptides can be visualized as horizontal bars positioned along the sequence of the protein.

Woods plots, volcano plots and Manhattan plots (examples are shown in Figure 8) are all valid options to show the results of a differential analysis. We find the Woods plots to be more *complete* as they show directly the difference in uptake and the position of the perturbation, and indirectly the statistical significance. Volcano plots show the difference in uptake and the statistical significance but fail to directly localize changes along the sequence of the protein; Manhattan plots can localize the differences and show the statistical significance but fail to show the difference in uptake.

#### 4.2.7 Which Statistical Test to Choose?

In section 4.2, we reviewed several strategies implemented in software to analyze differential HDX-MS data: (i) manual thresholding, (ii) simple hypothesis testing (*t* test), (iii) linear regression model, (iv) mixed models, and (iv) functional analysis-based strategies. We strongly suggest avoiding manual thresholding as it fails to control for false positives. While simple hypothesis testing is not inherently flawed, it can be easily generalized into a linear regression model. A *t* test is limited to comparing one explanatory variable at a time, whereas a linear model can account for multiple variables—such as labeling time, experimental condition, and charge state—making it more suitable for HDX-MS data. The choice between linear models, mixed models or functional analysis-based strategies depends on the data set available and on the specific research question. Mixed models are ideal in cases where data are not independent, such as when both biological and technical replicates are available, when meta-analysis of data sets from different research groups needs to be performed, or when newly acquired data on one variant of a protein have to be compared with older data, possibly collected by different researchers but using the same instrument. In most scenarios, a linear model is the most pragmatic solution to assess statistically significant differences between two conditions. The function analysis-based strategies, on the other hand, are powerful when kinetic information is needed, but require an informed selection of time points to accurately estimate the underlying deuterium uptake curve.

### 4.3 Multimodal Analysis

**Figure 9:**
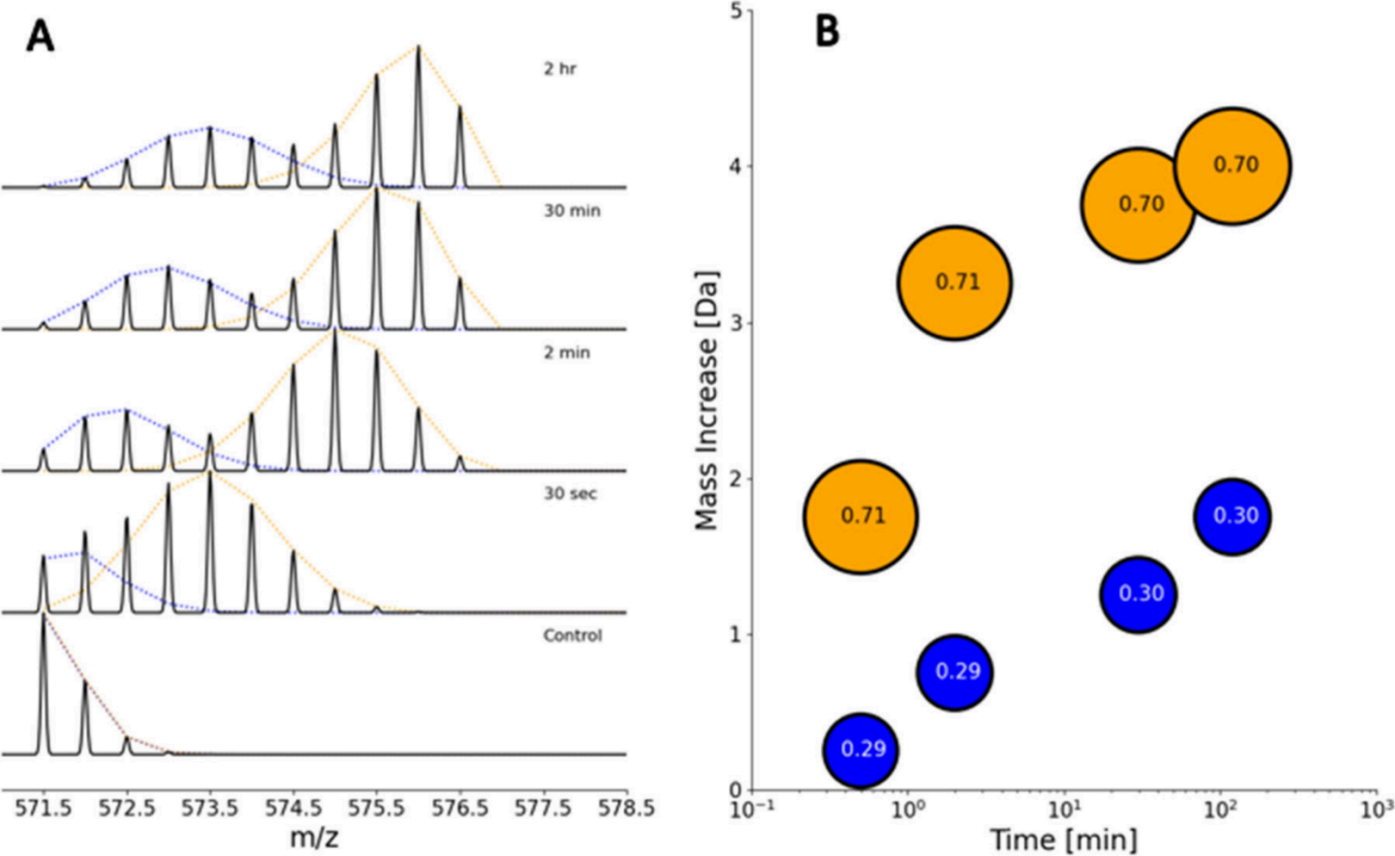
Multimodal behavior from two coexisting conformations. (A) The time evolution of the isotopic envelope of a peptide with bimodal behavior is shown. Both populations follow an EX2 kinetics, and therefore identify two distinct conformations of the protein that are not interexchanging. The bimodal distribution is fitted with two binomial distributions, and the mass increase and ratio of the two populations is recorded. (B) The mass increase of the two populations is shown as a function of time. The size of the scatter points is proportional to the fraction of molecules following the specific population.

Sometimes the presence of EX1, mixed EX1/EX2 (also known as EXX) behavior, or the coexistence of multiple conformational states of a protein, can cause the isotopic envelope to assume a multimodal shape (Figure 9). Pure EX1 kinetics can produce two isotopic envelopes with fixed *m*/*z* values (the fully protonated and fully deuterated) but with variable intensities (the intensity of the fully deuterated envelope increases and the undeuterated one decreases accordingly over time). The coexistence of EX1 and EX2 kinetics (EXX) is also characterized by the presence of bimodal isotopic envelopes, with the first population gradually shifting toward higher *m*/*z* values (as in the EX2 regime) and the second associated to the fully deuterated spectrum (as in a pure EX1 kinetics); in this mixed regime, an intensity shift to the higher-deuterated state is observed. It is also common to find a multimodal behavior with two populations that can both undergo EX2 kinetics, which is associated with two distinct conformations of the protein that are not interexchanging.^92,93^ In such cases, the modes of the bimodal spectrum should be deconvolved before comparing the intensity-weighted average of the individual populations with the statistical models described in section 4.2. In other words, two values of deuterium uptake are needed to fit the isotopic distribution properly and to quantify the fraction of molecules following EX1 or EX2 behavior (or, analogously, the population in either conformational state). Note that EX1 kinetics is a rare phenomenon and should not be confused with carryover.^94^

To perform a multimodal analysis, the raw mass spectra of the proteolytic peptides are needed in order to obtain the full isotopic distributions. We note here that retrieving such raw spectra is not trivial: the majority of the tools described here require the csv output files generated by DynamX (for Waters instruments) or HDExaminer (for Thermo Fisher Scientific), which only contain information on the intensity-weighted average of the isotopic envelope that have been automatically assigned and manually curated. Manually analyzing raw data is very time-consuming and error prone, even for a data set with a limited number of samples. One can also use tools from MS-based proteomics and/or MS-based metabolomics to extract the needed information from the raw spectra. However, these tools cannot be used out-of-the-box and are therefore not very user-friendly for the inexperienced user. A third option is to implement a method from scratch that takes as input the raw files, implements a peptide search, carries out automated or manual mass spectrum assignments, and stores information on the shape of the isotopic envelopes. The latter strategy has been developed by several groups which, being able to interface with raw data, have developed methods to study the bimodal behavior caused by EXX kinetics or by the coexistence of multiple conformations.

Mass Spec Studio^49^ and HX-Express,^41^ for example, can identify peptides showing bimodal behavior in the isotopic distribution. These software packages allow fitting experimental spectra with a double binomial distribution and to extract the associated parameters, namely the center of mass of the two subdistributions and their relative intensities.

ExMS2^36^ can detect peptides showing multimodal behavior through a “unimodality check” introduced in the latest version of the software to assess the quality of peptide selection. These peptides are flagged and can be further studied by a module named “Multimodal analysis”. The isotopic envelope of the peptide can be fitted with several functions (varied binomials, uniform binomial and Gaussian(s), Gaussians, or reference shapes—in case a control sample displaying the pure subspectrum of one population is available). The multimodal behavior can be detected, and the parameters extracted through the fitting procedure can be used to determine the fraction of sample following EX1 or EX2 regime.

deMix^33^ is a recent method aiming to tackle the issue of discriminating different populations when a bimodal distribution appears due to mixed EX1 and EX2 behavior in HDX-MS data. The deuterated isotopic distribution (of every peptide and at every time point) is fitted with a separate binomial distribution. An optimal value for deuteration *d*_A_ is calculated. If the deuterated distribution is not explained enough by *d*_A_, then bimodal analysis is performed. The top two-scoring deuteration values *d*_A_ and *d*_B_ are calculated. The resulting bimodal distribution is fitted with experimental data to determine how each species is populated. deMix reports two values of deuteration only if the error of the bimodal distribution is significantly improved and if the weight factor for the least abundant species is greater than 10%.

Here we presented various strategies for analyzing HDX-MS data of peptides exhibiting multimodal behavior, which can arise from several factors, including carryover, coexistence of different noninteracting protein conformations, EX1 kinetics, or mixed EXX kinetics. The methods discussed here enable robust deconvolution of the extent of deuteration of each population, but they do not inform the user per se about what causes the bimodal behavior. If bimodality is known to be due to carryover, then these methods allow for its correction (rather than redoing the experiment). In the case of coexisting protein conformations (both following EX2 kinetics), they help determining the fraction of molecules in each conformation. When the relative intensities of both populations are sufficiently high, this enables the study of the exchange kinetics of both conformations. In the rare instances of pure EX1 kinetics or mixed EX1/EX2 kinetics, these methods allow determination of the fraction of fast- and slow-exchanging molecules. The weakness in the latter scenario is the unclear application of this information. Indeed, peptides showing EX1 or EXX kinetics are generally excluded from differential analysis. Sometimes, for example, standard EX2 kinetics might be observed for one protein state, while pure EX1 kinetics is observed for another. It is true that the emergence of EX1 or mixed EXX kinetics can qualitatively assess protein disorder, but quantitatively assess statistically significant differences between different states and integrating these data into modeling remain unresolved challenges.

### 4.4 Protection Factor Analysis

The Linderstrøm-Lang model (eq 1) describes HDX as a phenomenon occurring at the level of the single residue. The exchange kinetics follows an exponential law with an exponent that, in the EX2 limit, depends on the intrinsic exchange rate and on the protection factor (eq 2). The intrinsic exchange rate represents the rate that the same type of residue (amino acid) has in a completely unfolded structure. The protection factor of the residue depends on the local structure of the protein surrounding the residue. Retrieving protection factors from HDX-MS data would enable to connect the experimental data with microscopic properties that can be inferred from atomistic modeling and MD simulations. Indeed, protection factors can be measured for labeled residues of a protein through HDX-NMR.^8^ However, the information provided by HDX-MS is coarse-grained to the peptide level and underdetermined (see section 3.5), and extracting protection factors (or exchange rates) at the resolution of the single amide from HDX-MS data is not trivial.

The spatial resolution of HDX-MS data can be increased experimentally. On the one hand, different proteases^20−23^ or multienzyme strategies have shown to be beneficial in increasing peptide overlaps.^95^ On the other hand, MS/MS fragment data can be exploited. Among the fragmentation techniques available, collision induced dissociation (CID) has the drawback of favoring H/D scrambling within the peptide (protons and deuterium atoms are mobile within the peptide). Alternative dissociation techniques, such as ECD/ETD (more generally ExD) and UVPD, have been proven to increase spatial resolution while minimizing H/D scrambling.^24,25^ However, reaching single residue resolution for the whole protein with these fragmentation techniques is still challenging, mainly for two reasons: sensitivity (the intensity of peptides and fragments vary significantly due to the broad specificity of pepsin, the fragmentation and ESI efficiencies) and protein size (the proportion of inter-residue cleavages decreases with the protein size).^96^

Advanced data analysis strategies can be used to computationally increase spatial resolution of peptide-level HDX-MS data or to estimate protection factors (computational tools for such purpose are listed in Table 3).

**Table 3:** List of Software Packages for High-Resolution HDX-MS Data Analysis at the Peptide Level

High Resolution HDX-MS	
Name	Strategy
DECA^32^	overlapping peptide segmentation
HDflex^42^	stretched exponential
Hdxstats^45^	stretched exponential
ExMS2^35,36^	isotopic envelope fitting
ExPfact^37,38^	intensity-weighted average fitting
pyHDX^52^	intensity-weighted average fitting
HDXModeller^44^	intensity-weighted average fitting
Saltzberg et al.^54^	intensity-weighted average fitting
HR-HDXMS^46^	intensity-weighted average fitting
ReX^53^	change-point model

**Figure 10:**
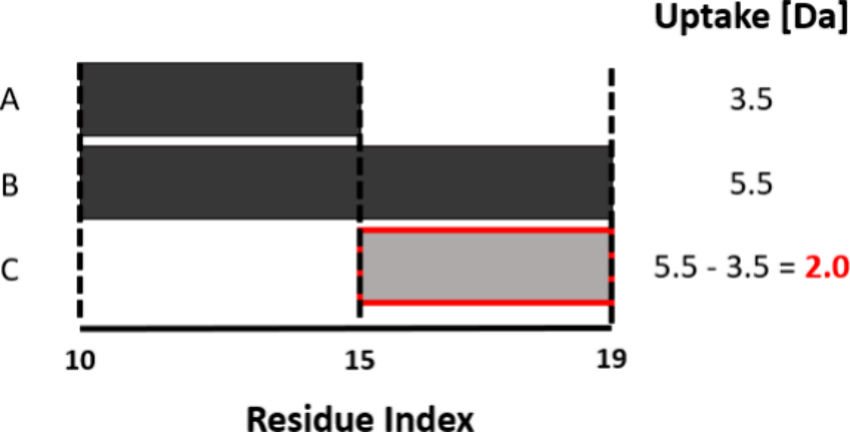
Example of Overlapping Peptide Segmentation (OPS) for two peptides A and B, covering respectively residues 10–15 and 10–19 and with absolute uptake 3.5 and 5.5 Da. OPS generates an artificial peptide C covering residues 15–19 with absolute uptake 2.0 Da.

To increase resolution, DECA^32^ implements a computational method named Overlapping Peptide Segmentation (OPS). OPS exploits the overlapping of peptides to assign better-resolved uptake values to nonoverlapping areas (Figure 10). When two peptides have a common terminus (e.g., peptide A covering residues 10–15 and peptide B covering residues 10–19), the absolute uptake of a smaller peptide defined by the nonoverlapping residues of the observed peptides (i.e., an artificial peptide C covering residues 16–19) is calculated as the difference in absolute uptake of the bigger peptides (if peptide A has absolute uptake 3.5 Da and peptide B 5.5 Da, the uptake of peptide C is set to 5.5–3.5 = 2.0 Da). Because of error propagation, the error associated with the uptake of these artificial peptides is bigger than the original. For this reason, OPS should not be repeated more than once.

HDfleX^42^ and hdxstats^45^ fit peptide level data with a stretched exponential (eq 12). The fit returns a peptide-level exchange rate that can be used to obtain a pseudo (peptide-level) protection factor. HDflex^42^ has the unique capability of analyzing peptide- and ETD fragment- level data simultaneously. The uptake curve of the peptide/fragment is divided by the number of exchangeable sites, so that the uptake curve of a residue is an average over the available peptides and fragments covering that specific residue. The combination of ETD data and this “data flattening” procedure thus allows an improvement in spatial resolution beyond the peptide level.

ExMS2^36^ contains a module named HDSite to extract protection factors. Here, the estimation of amide exchange rates can be performed using two different strategies. In the envelope-based method, the isotopic envelopes calculated by ExMS2 are fitted at each time point to calculate the deuteration of the residues, exploiting the overlapping of peptides. The uptake of each residue is then fitted with a single exponential (eq 2) to extract the exchange rate of the single amide. Alternatively, HDSite attempts to directly fit the amide exchange rates for a whole set of peptides and exchange times. These two methods provide better results depending on the data set.

ExPfact^37^ is a computational method aiming to extract protection factors at the resolution of the single amide and relies on the information encoded in the intensity-weighted average of the isotopic envelopes. The time-dependent uptake of each peptide is fitted simultaneously with eq 7 and the values of the protection factors are adjusted to minimize the difference between predicted and experimental values (i.e., a cost function). Because of underdetermination, the solution is not unique (the existence of a multiplicity of solutions is known as “degeneracy”): different sets of protection factors have the same agreement with experimental data. To attenuate the degeneracy of the solutions, the fitting algorithm is coupled to a *regularizer*, i.e. an additional term in the cost function that favors the finding of smooth patterns of protection factors (this can be interpreted as an assumption that adjacent residues do not “jump” in protection). ExPfact calculates alternative sets of protection factors, where each set is the result of a minimization procedure starting from a randomized initial guess. To further reduce the degeneracy of the solutions, a clustering algorithm (based on a mixture of multivariate Gaussian distributions) is applied and ExPfact returns a discrete number of families of solutions. Each element of each family is a set of protection factors in agreement with experimental data. The additional information contained in the isotopic distribution can be used a posteriori to rank sets of protection factors.^38^ HDSite and ExPfact have been cross-validated with HDX-NMR data.

HDXModeller^44^ implements a strategy very similar to ExPfact: a minimization procedure is repeated multiple times starting from a random initial guess; the software introduces a correlation matrix as an autovalidation tool to estimate the accuracy of the modeled protection factor of individual amino acids.

PyHDX^52^ uses a machine learning framework to perform the fitting directly in a free energy landscape (the connection between the protection factor and the free energy is *P* = e^Δ*G*/*RT*^, where Δ*G* represents the difference in free energy between the open and closed states); the problem of underdetermination is mitigated because one specific initial guess is selected and the (stochastic) fitting algorithm is coupled with a regularizer.

Also HR-HDXMS^46^ implements nonlinear programming to estimate HDX exchange rates at single amino acid resolution. The degeneracy is moderated by choosing a data-oriented initial guess for the exchange rates: Overlapping Peptide Segmentation (OPS) is used to artificially increase spatial resolution and the deuteration of subpeptides is fitted with an exponential model to obtain a rate constant. This rate constant is used as initial guess for all the residues belonging to the subpeptide considered.

A Bayesian framework to estimate protection factors from HDX-MS data was first proposed by Saltzberg et al.^54^ The Bayesian approach estimates the probability of a particular model, given all the information about the modeled system, including prior knowledge of the system, experimental data on the system and models of experimental noise. In other words, the output of a Bayesian approach is the probability distribution of an exchange rate is calculated, not a specific value. The problem of selecting an initial guess is translated into selecting an initial probability distribution. An uninformative Jeffrey’s prior (which corresponds to a uniform probability distribution) is applied to each individual exchange rate constant to represent a lack of information on the bounds and distribution of the parameter. Best scoring solutions are clustered, and mean values and standard deviations are reported.

ReX^53^ is a new strategy, proposed by the same authors of Hdxstats,^45^ to infer residue-level rates from HDX-MS data. “ReX combines a likelihood model, which models the deuterium per residue, with a prior change-point model that permits correlations or jumps between the parameters of adjacent residues”.^53^ HDX is modeled as a latent process (i.e., unobserved) occurring at the level of the single amino acid. The exchange of each residue is modeled as a mixture of a stretched exponential (eq 12) and a standard exponential (eq 2)—the proportion of the mixture is learned during the inference process. If every residue was considered as a separate entity, then the model would have too many parameters to be fitted to experimental data. To overcome this issue, a change-point model^97,98^ is implemented, which allows the parameters between segments of residues be either similar or discontinuous (jump). The number of change points (*where* the jump occurs) is determined via a specific Markov Chain Monte Carlo (MCMC) algorithm, known as Reverse Jump Markov Chain Monte Carlo (RJMCMC),^97^ that allows the number of change points to be variable (i.e., not fixed a priori).

Protection factor analysis requires knowledge of the contributions of each amide to the overall, observed deuterium incorporation into a peptide. In this section, we reviewed various strategies developed to increase spatial resolution from peptide-level HDX-MS data, which can be grouped into five classes (Table 3): (i) overlapping peptide segmentation, (ii) stretched exponential, (iii) isotopic envelope fitting, (iv) intensity-weighted average fitting, and (v) change-point model. We discourage the use of overlapping peptide segmentation as it has been shown that subtractive methods for improving spatial resolution in HDX-MS data often yield inaccurate predictions as they neglect different levels of back-exchange for peptides of different lengths.^70^ Fitting individual uptake curves with a stretched exponential can be useful to obtain a qualitative parameter describing the kinetics of a specific peptide, but this parameter is barely connected with the parameters of the Linderstrøm-Lang model (opening/closing rate or protection factor). The same limitation applies to the change-point model. We believe the most effective strategies to achieve single-residue resolution from peptide-level experimental data are the isotopic envelope fitting provided by ExMS2 and the intensity-weighted average fitting provided by ExPfact. These are the only two methods that have been cross-validated with NMR experiments, demonstrating a strong correlation between the protection factors derived from both techniques. A reference data set analyzing the HDX of a model protein with both NMR and MS would significantly aid the development of these methods. The main drawback associated with these strategies is that the results are highly dependent on the quality of the HDX-MS data set, which is determined by the number of peptides and redundancy provided by the coverage map, as well as by the number and distribution of labeling time points. Additionally, the limited understanding of back-exchange and of EX1/EXX kinetics are holding back the development of these methods, which remain an active area of research. While they have shown promising results in inferring single residue resolution from peptide level data, a protocol to perform a “protection factor analysis” for HDX-MS data has yet to be established. To encourage the use of the tools described here across the community, software developers should prioritize the creation of user-friendly graphical interfaces, comprehensive documentation, and tutorials.

## 5 Concluding Remarks

The growing popularity of HDX-MS spurred the recent development of several data analysis tools, which are described here alongside more basic (vendor-specific) software. We took the different steps of the data analysis workflow of HDX-MS as a guide and discussed how the preprocessing of raw data, which is generally performed with vendor-specific software, can be now performed with alternative open-source platforms, allowing the user to better interact with the raw data. Moreover, commercial software such as Protein Metrics (Dotmatics) and HDX Workbench^27^ provide a comprehensive tool for HDX-MS data analysis. The curation of HDX-MS is however still lacking in some aspects of a complete theoretical understanding, for example in a proper correction for back-exchange.

We discussed differential experiments, where HDX-MS enables the relative and qualitative comparison of exchange patterns under different experimental conditions to pinpoint perturbations along a protein’s sequence. While statistical analysis and data visualization for differential HDX-MS experiments are now well-established, there are still some nuanced aspects that warrant attention. First and foremost is the critical choice of an appropriate statistical test for comparing exchange curves across different states. We advocate for the use of statistical tests (*t* tests, functional analysis, linear models, or mixed models) over manual thresholding. The rationale behind this choice is that the latter approach provides no control over false positives and false negatives. Additionally, we encourage the use of multiple testing corrections. The selection of the most appropriate statistical test is contingent upon the experimental design’s architecture. For experiments encompassing both technical and biological replicates, mixed models emerge as the optimal choice. Conversely, if only one type of replication is available and no specific information about the average exchange rate is required, then the linear model represents the simplest and most pragmatic alternative. Functional analysis offers the advantage of modeling the time variable and providing quantitative insights into exchange kinetics at the cost of needing many time points to model the nonlinear relation adequately. Second, it is worth noting the well-established observation that the deuteration of a peptide can be influenced by its charge state. This phenomenon, which arises from back-exchange occurring during the gas phase, remains incompletely understood, and necessitates a careful treatment of different charge states to avert spurious discoveries. When performing a differential analysis, it is important to compare the same charge state for the different experimental conditions available. When multiple charge states have been detected, it is important to check that the same results (protection/deprotection) are consistent among the different charge states. This can relatively easily be tackled by adding an extra variable (the charge state) to the linear model implemented in eq 9.

We discussed how conventional differential analysis approaches should be coupled with deconvolution tools when dealing with peptides exhibiting EX1 or EXX kinetics. Several methodologies have been devised to address these scenarios, enabling the analysis of the bimodal behavior of isotopic envelopes and the extraction of information regarding deuteration and the fractions of the two modes involved. However, it is important to acknowledge that such analysis hinges on the availability of raw mass spectra, which can be challenging to obtain. Moreover, it remains unclear how the information derived from bimodal distributions can be interpreted and integrated with protein modeling, and care should be taken when coming across such complex peptide spectra, and their precise cause established. We also discussed how HDX-MS holds promise beyond its utility in differential experiments. It affords the opportunity to delve into exchange kinetics at the single-residue level, making it an ideal candidate for validating ab initio models or predictions of protein structure. Numerous techniques have been proposed for extracting protection factors from HDX-MS data, but a universally accepted standard for protection factor analysis has yet to be established.

What should ideal HDX-MS software look like in five years’ time? Ultimately, the goal of HDX-MS software development is to enable researchers to obtain a deeper understanding of protein dynamics, functions and interactions. Therefore, the software should remove HDX-MS experimental idiosyncrasies and express the information contained in the data in the form of more physical descriptions of protein dynamics. These physical descriptors can take many forms, for example as outputs which are already established, such as protection factors or Gibbs free energies. Due to the richness of HDX-MS data sets we anticipate that future software development can give more detailed insights into hydrogen-bond networks and protein allostery, identify regions of local cooperative unfolding, or generalize functional patterns from series of protein mutations. These physical descriptions of protein dynamics could then function as input for downstream bioinformatics methods, in the form of constraints for molecular dynamics simulations or as training data for predictive artificial intelligence (AI), taking deep learning approaches such as Alphafold^99^ beyond static predictions of protein structure and instead offer functional information based on protein dynamics. For example, predictive AI models could learn from HDX-MS data how to identify allosteric regulation in de novo designed proteins.

In general, while it is important that software serves the direct needs of the HDX-MS community itself, in the form of statistical testing and data set quality validation, we envision that future software development will facilitate dissemination of novel insights toward broader audiences and allow for increased interfacing with neighboring fields.

To work toward these goals, the software should perform the following basic steps. The software would accept the protein sequence and undeuterated raw data as input, performing robust peptide search and identification to generate a coverage map. This search would support not only peptide-level data but also fragment-level data from CID, ExD, or UVPD fragmentation. In the next step, the software would process the deuterated raw data, automatically detecting the isotopic envelopes of previously identified peptides. Since a large body of user-annotated peptides data sets are readily available, we anticipate that AI models can be trained on this data and provide further automation and validation in this critical step, increasing both throughput and accuracy. The identified peptides could then be exported in a single operation as isotopic envelopes in a standardized format. It would also perform accurate back-exchange correction (or the best available correction based on future research), showing users how the correction modifies raw input data and provide feedback on confidence and potential experimental artifacts. The software would deconvolute peptide spectra exhibiting multimodal behavior, enabling researchers to export results for further analysis of EX1/EXX kinetics.

In “Differential Analysis” mode, researchers could select the most appropriate statistical test for their experimental design and research question. The software would then generate publication-quality Woods plots, Manhattan plots, and volcano plots to highlight statistically significant changes across the protein sequence. Protein structural information could be uploaded to the software, either obtained from experimental methods or Alphafold predictions. The software could feature one or multiple modeling options or fitting strategies, such as “Protection Factor Analysis”, where users are guided through steps and various modeling parameters, and the software would evaluate the data set’s quality and providing a confidence level for the final predictions. The estimated pattern of protection factors or other modeling output could then be mapped onto the uploaded protein structure and presented as an integrative structural and functional output. There should be a strong focus on accessibility, providing comprehensive documentation and a user-friendly graphical user interface. Data processing best-practice and the effect of user-configurable settings and tunable parameters such as thresholds and how they influence output and confidence should be clearly explained through tutorials or other forms of documentation. Publication of source code under a permissive license is required for other researchers to validate and review the processing pipelines as well collaborate and iterate on published works.

Experimental researchers would focus on the experiment, and the software would provide real-time results and suggestions to guide their decisions, while computational researchers would be able to download online data sets from a standardized repository, rapidly perform the same analysis performed in published papers, and easily and exhaustively export all the information they need to improve the implemented methods or to propose alternative solutions to tackle the remaining challenges.
